# Supplementary material for: The role of cell geometry and cell-cell communication in gradient sensing
Source: PLoS Comput Biol. 2022 Mar 14;18(3):e1009552. doi: 10.1371/journal.pcbi.1009552 (PMC8963572; doi:10.1371/journal.pcbi.1009552)
Supplement: S1 Text — (PDF) [file pcbi.1009552.s001.pdf]

**S1 text  Including global reporter escape in the ISD model.**

Here we extend the analytical calculations in the ISD model to the case in which global reporter molecules can escape the lateral intercellular space (LIS). In particular, we study how the escape process affects the probability of exchange of reporter molecules between neighbouring cells of different order.

We recall the expressions of the probability that the molecule is internalized at a time $t < \tau$ in the original calculation (Section 3.1.1)

$$P(0 < t < \tau) = \int_0^\tau \lambda e^{-\lambda t} dt = 1 - e^{-\lambda \tau} \quad , \tag{1}$$

and the probability that the molecule is internalized before travelling a distance $s$

$$P_{int}(s) = 1 - e^{-\sqrt{\frac{\lambda s^2}{D}}} \quad . \tag{2}$$

Assuming that molecule escape is described by an exponential decay, the probability of not being escaped before a time $t$ is $e^{-\nu\tau}$, where $\nu$ is the escape rate. Moreover, assuming that molecular escape and internalization are independent processes, Eq (1) now reads

$$P(0 < t < \tau) = \int_0^\tau \lambda e^{-\lambda t} e^{-\nu t} dt = \frac{\lambda}{\lambda'} \left(1 - e^{-\lambda' \tau}\right) \quad , \tag{3}$$

where $\lambda' = \lambda + \nu$.

In turn, the probability that the molecule is internalized without escaping before travelling a distance $s$, which we indicate as $\tilde{P}_{int}(s)$, is

$$\tilde{P}_{int}(s) = \frac{\lambda}{\lambda'} \left(1 - e^{-\sqrt{\frac{\lambda' s^2}{D}}}\right) \quad . \tag{4}$$

From Eqs (2) and (4) we can obtain the probability that a global reporter secreted by cell $n$ is internalized by cell $n'$, taking into account the probability that the global reporter has diffused out of the LIS:

$$P_{nn'} \sim \frac{\tilde{P}_{int}(2l_E)}{N_m} \left[1 - P_{int}(2(m-1)l_E)\right] \left[1 - P_{esc}(2(m-1)l_E)\right] \quad , \tag{5}$$

where $n$ and $n'$ are neighbours of order $m$ (i.e., $m = 1$ corresponds to nearest neighbours), $l_E$ is the average edge length and $N_m$ is the number of neighbours of cell $n$ of order $m$.

The first term represents the probability that the molecule is internalized without escaping before travelling the last 2 edges; the second one is the probability of no internalization before travelling the first $m-1$ neighbour cell boundaries (i.e., a distance $2(m-1)l_E$); the third term represents the probability of not being escaped before travelling the distance $2(m-1)l_E$.

The probability that the molecule escaped before travelling a distance $2(m-1)l_E$ is obtained as $P_{int}$

$$P_{esc}(2(m-1)l_E) = 1 - e^{-\sqrt{\nu[2(m-1)l_E]^2/D}} \quad . \tag{6}$$

The series expansion of Eq (5) for $\nu \sim 0$, written in terms of original exchange probability (i.e., without considering the escape process), is given by

$$P_{nn'} = P_{nn'}(\nu = 0) \left[1 - \sqrt{\frac{[2(m-1)l_E]^2}{D}} \sqrt{\nu}\right] + O(\nu) \quad . \tag{7}$$

In S1 Fig, we set $\lambda = 1.0\ s^{-1}$, $D = 10\ \mu m^2/s$ or $D = 1000\ \mu m^2/s$ as in the other analyses, $l_E = 7.1\ \mu m$ and $N_m = 6$ or $N_m = 5$. We plot $P_{int}(s)$ as a function of the distance $s$ ($0 < s < 4 \cdot 2l_E$, to consider distances until neighbours of order 4) and $P_{nn'}$ as a function of the order of neighbours $m$ in the case of local and global communication. The results show that an escape rate $\nu$ such that $\nu/\lambda \lesssim 0.2$ has very little effect on the probability of exchange of the global reporter molecule between cells.
