# Supplementary figures and images for: The role of cell geometry and cell-cell communication in gradient sensing
Source: PLoS Comput Biol. 2022 Mar 14;18(3):e1009552. doi: 10.1371/journal.pcbi.1009552 (PMC8963572; doi:10.1371/journal.pcbi.1009552)

**A**

$$\lambda = 1.0 \text{ s}^{-1}; l_E = 7.1 \text{ } \mu\text{m}; N_m = 6$$

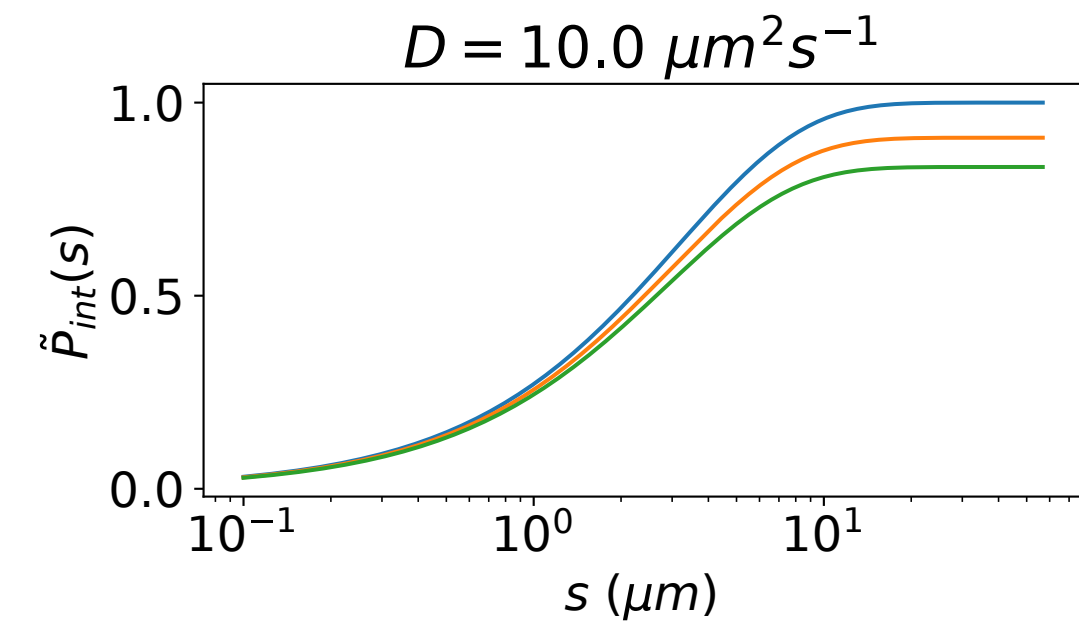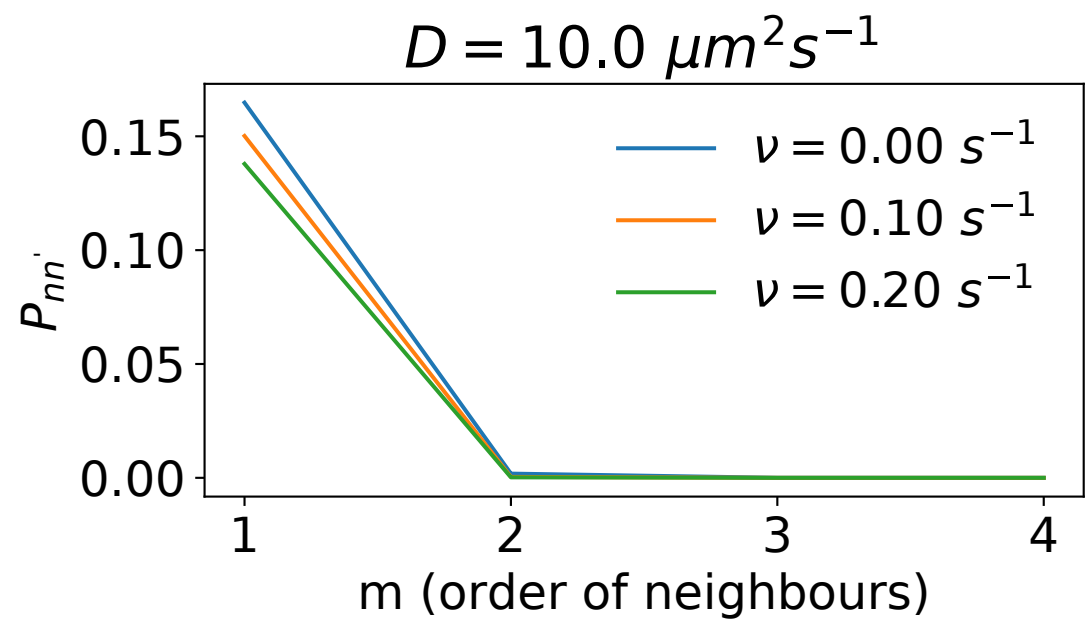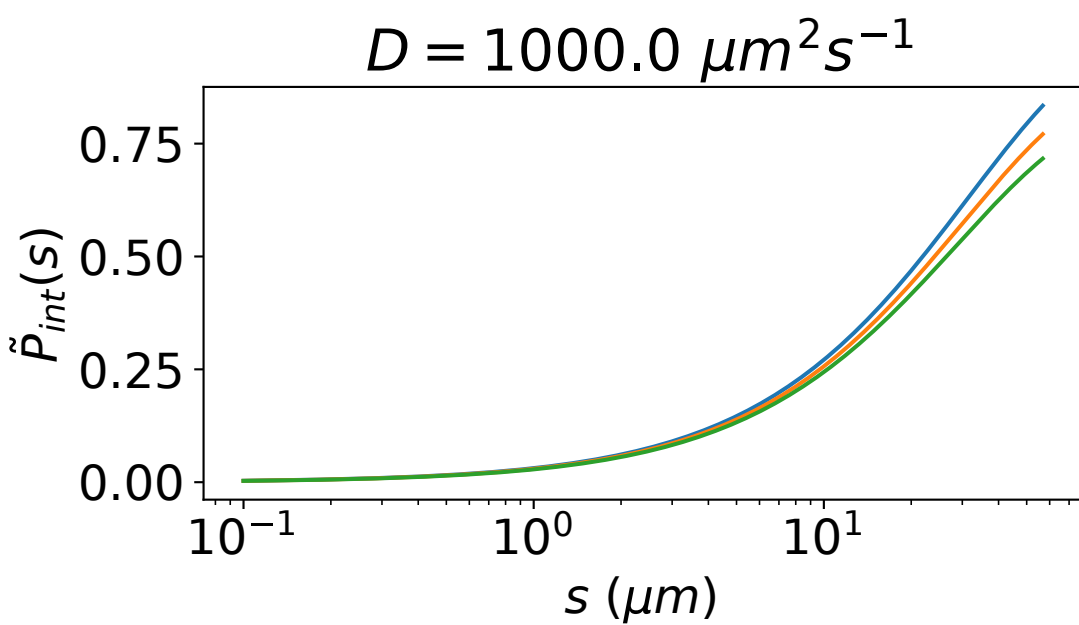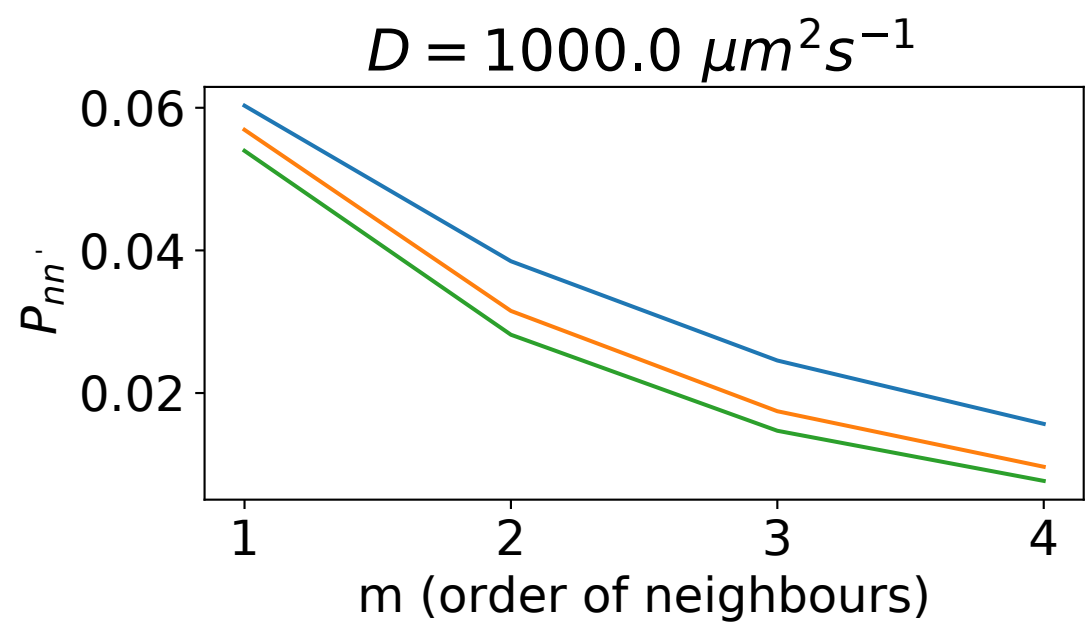**B**

$$\lambda = 1.0 \text{ s}^{-1}; l_E = 7.1 \text{ } \mu\text{m}; N_m = 5$$

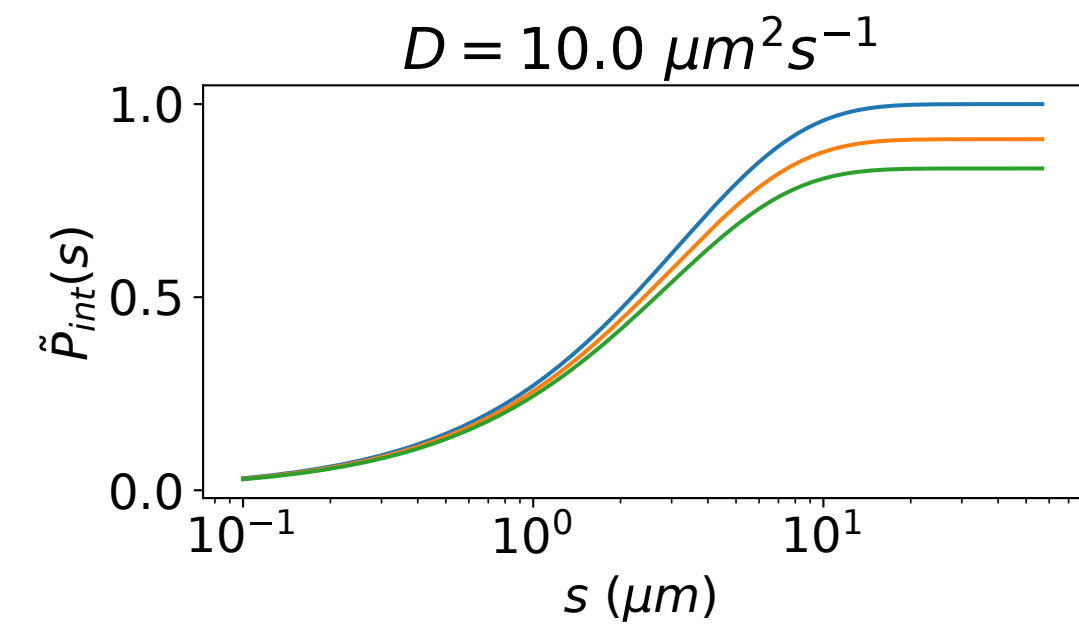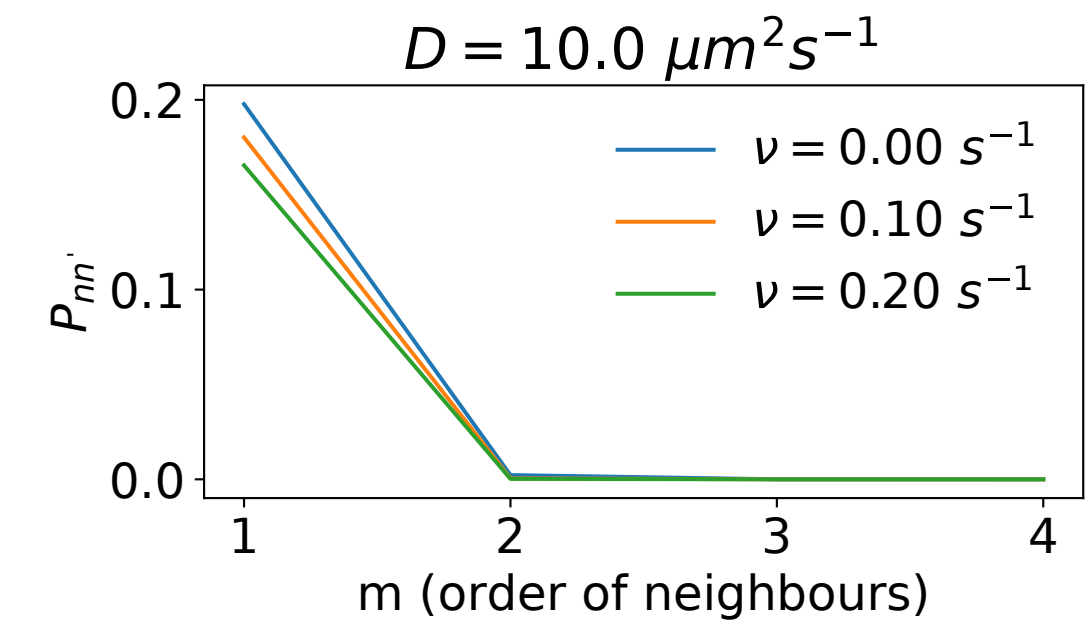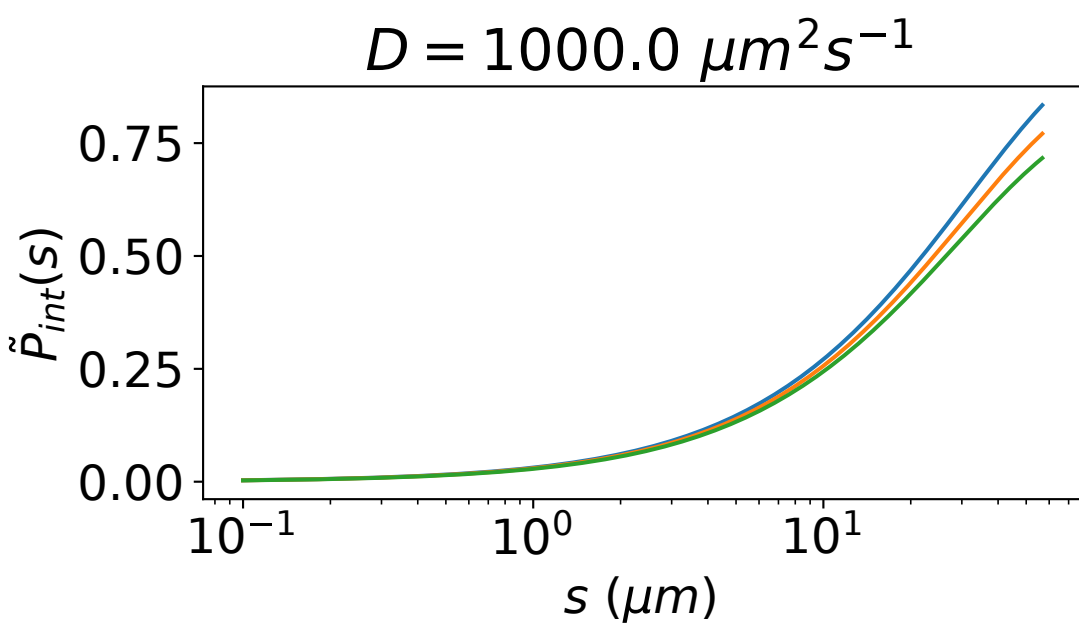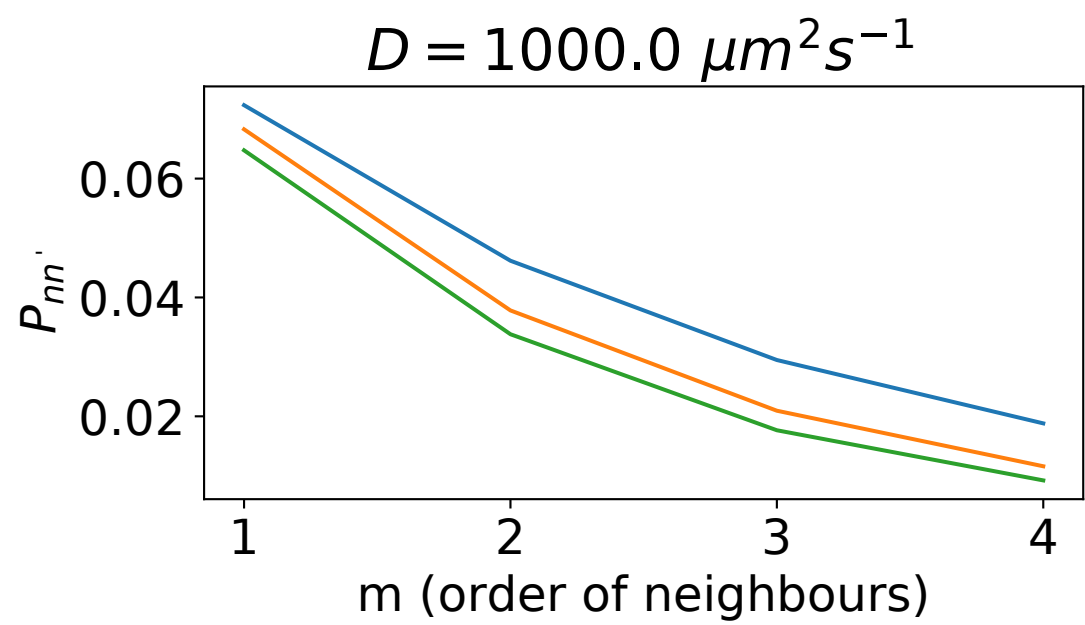

Supplement: S1 Fig — Probability of reporter internalization before travelling a distance s P˜int(s) (left), and probability of exchange of the reporter between a pair of neighbouring cells of order m, Pnn′, for different values of the escape rate ν and the two values of the reporter diffusion coefficients. Nm is the number of neighbours of the cell releasing the global reporter. A: Nm = 6. B: Nm = 5. Other parameters are indicated at the top of each panel. See S1 Text for the mathematical details. (PDF) [file pcbi.1009552.s002.pdf]

**A**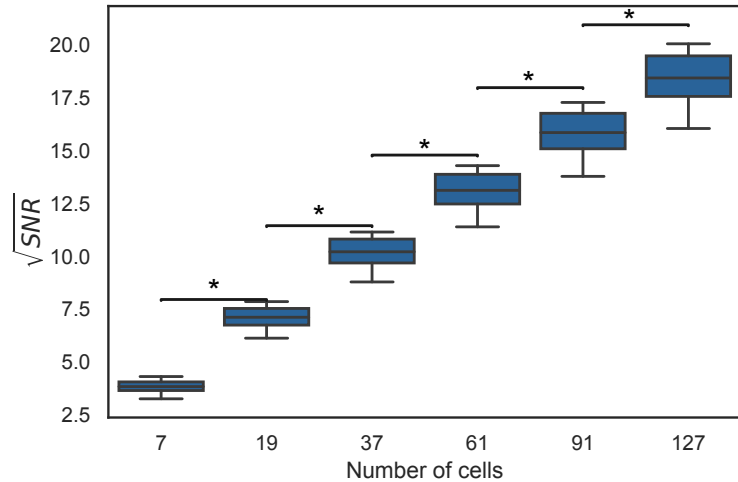**B**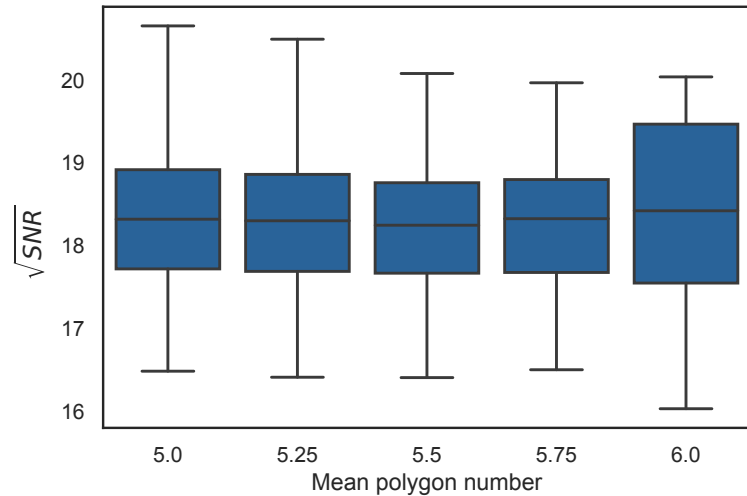

Supplement: S2 Fig — A: Box plots comparing the set of SNR values for configurations with different number of cells and mean polygon number 6. B: Box plots comparing the set of SNR values for configurations with different values of the mean polygon number and 127 cells. D = 1000.0 μm2/s, α = 100.0 s−1. The ⋆ indicates statistically significant results (see Materials and methods for further details). (PDF) [file pcbi.1009552.s003.pdf]

A

Weak - local (ISD)

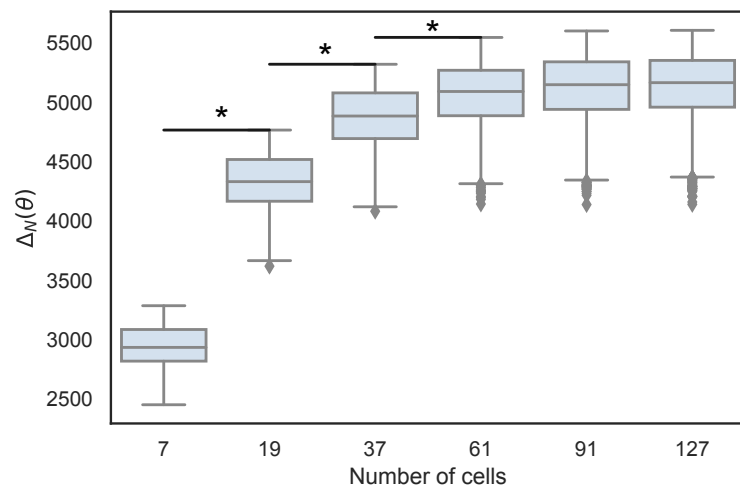

B

Weak (NNE)

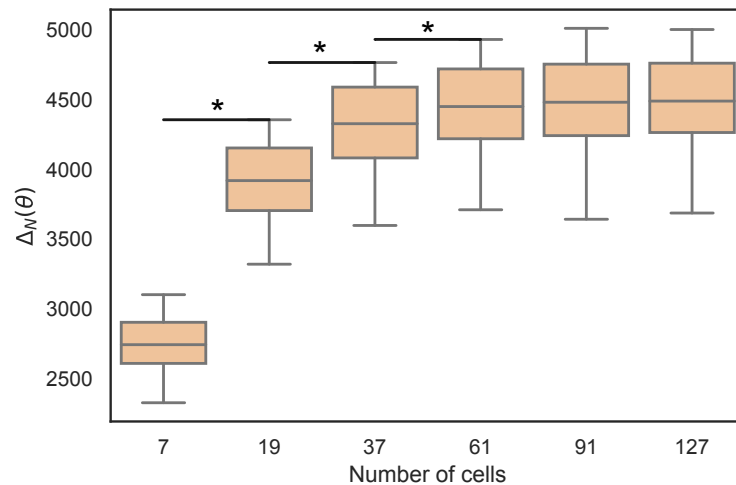

C

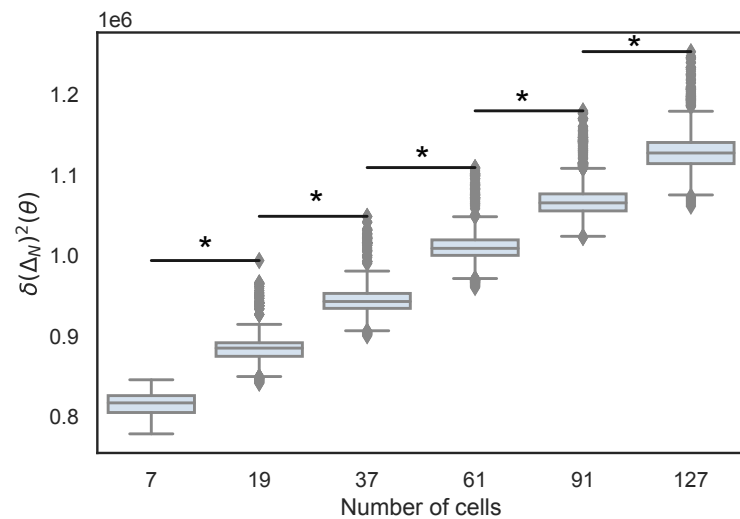

D

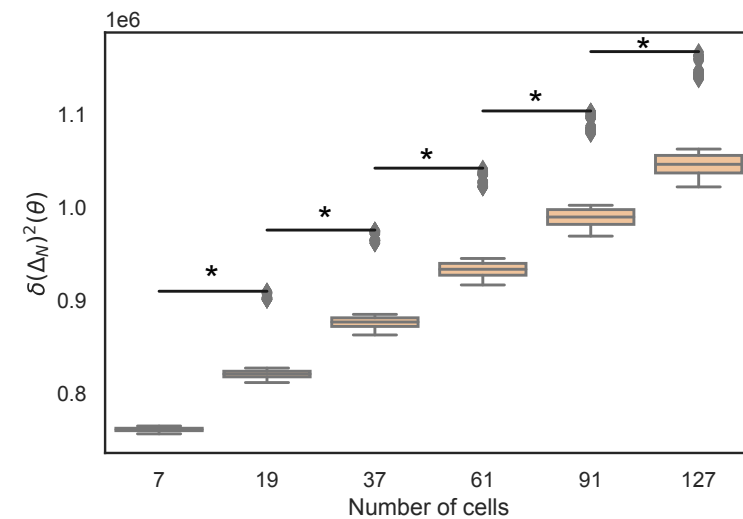

Supplement: S3 Fig — Mean (panels A-B) and variance (panels C-D) of the LEGI readout variable (Eqs (5) and (6)) in the weak-local (ISD) and weak (NNE) communication regimes, for the same configurations shown in Fig 5. (PDF) [file pcbi.1009552.s004.pdf]

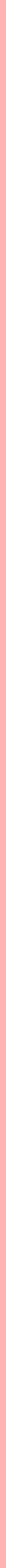

# NNE

## Strong-local

## Strong-global

Strong

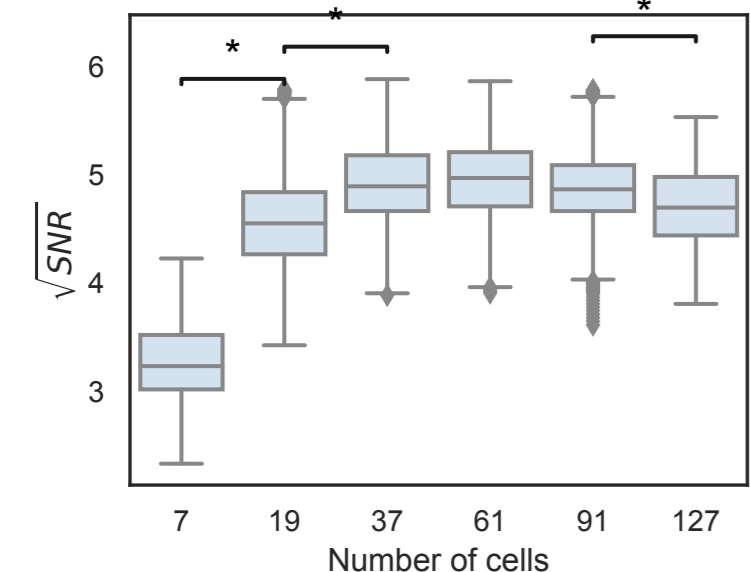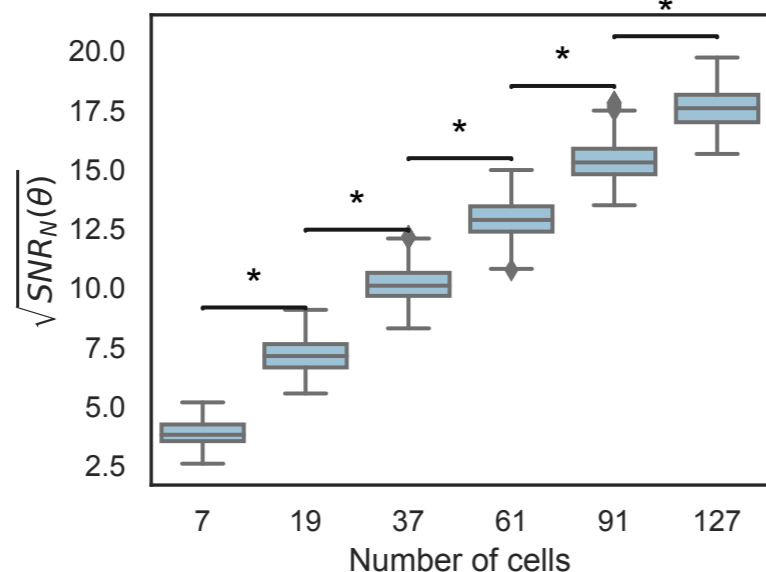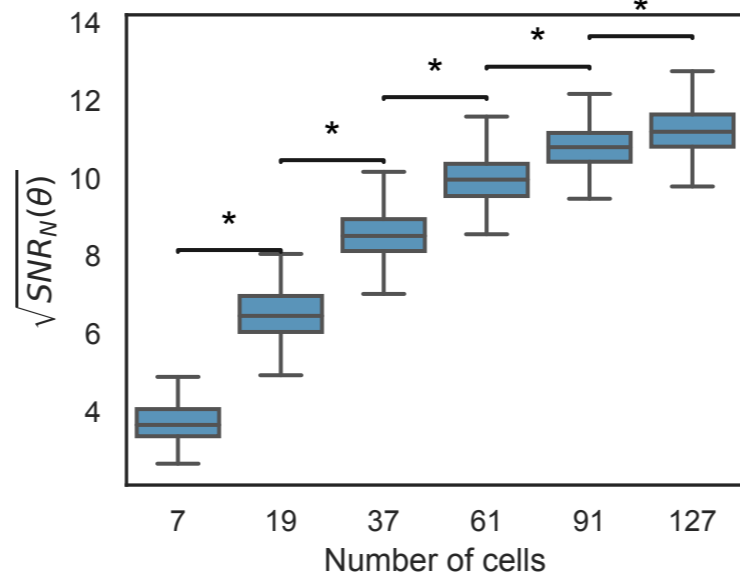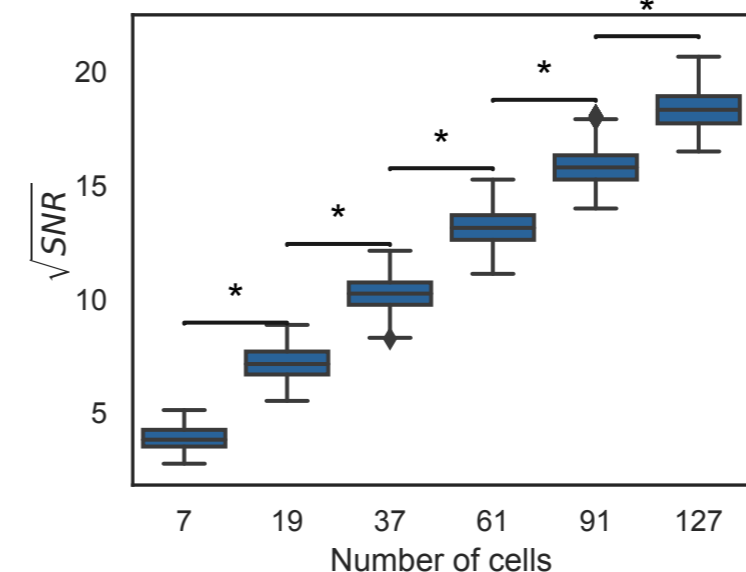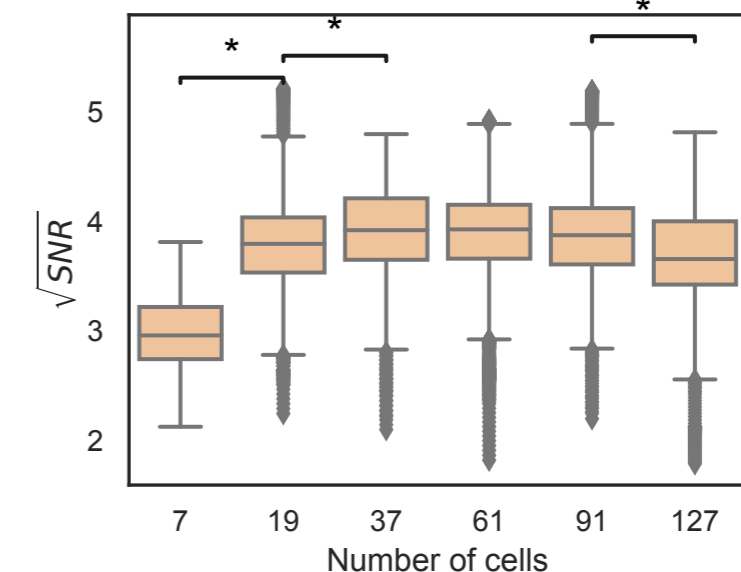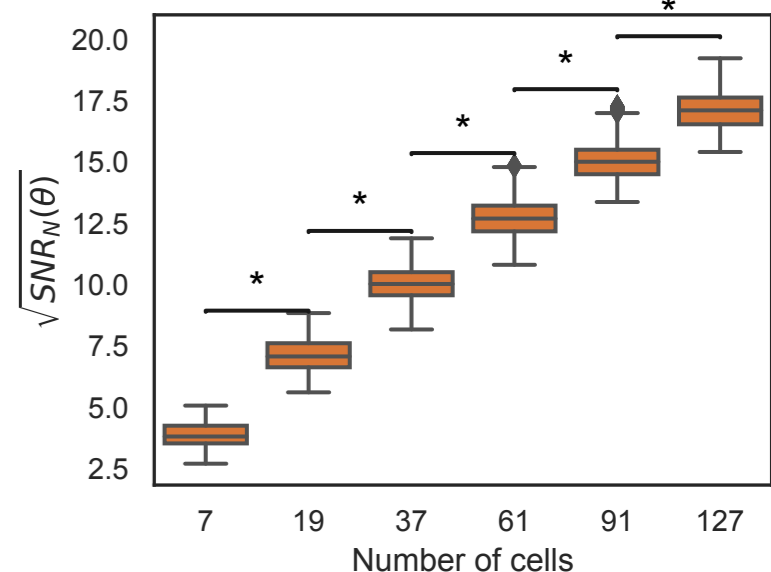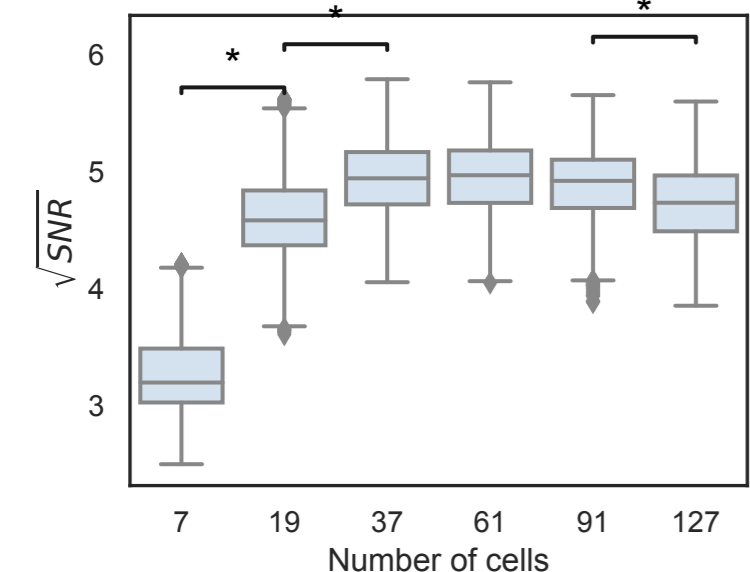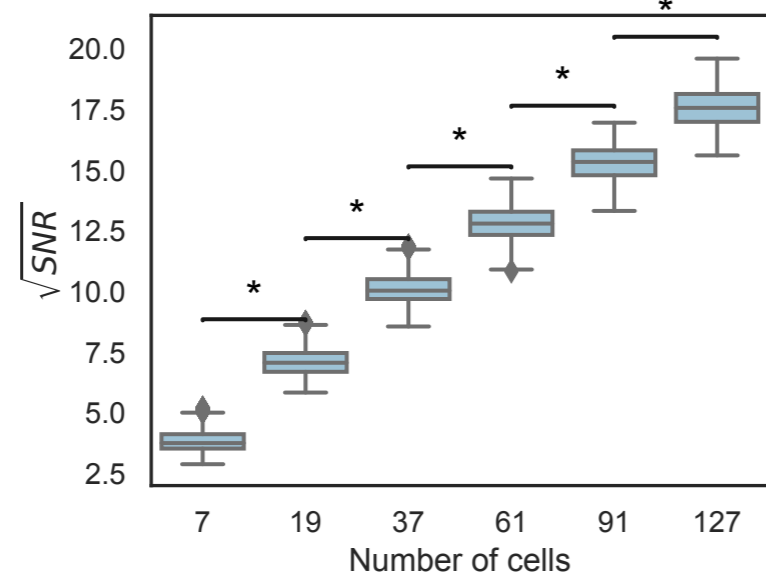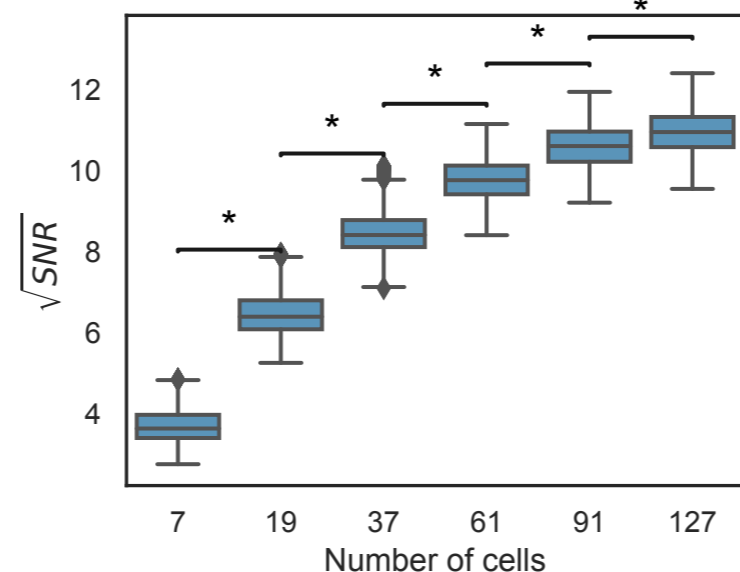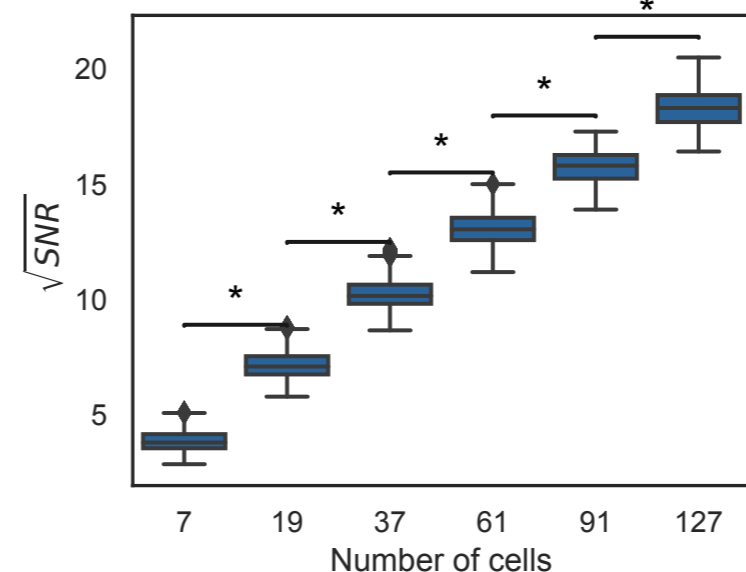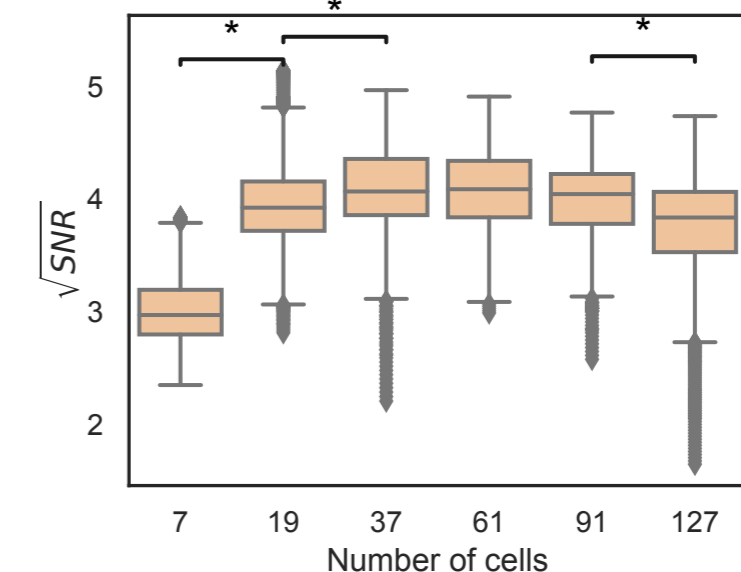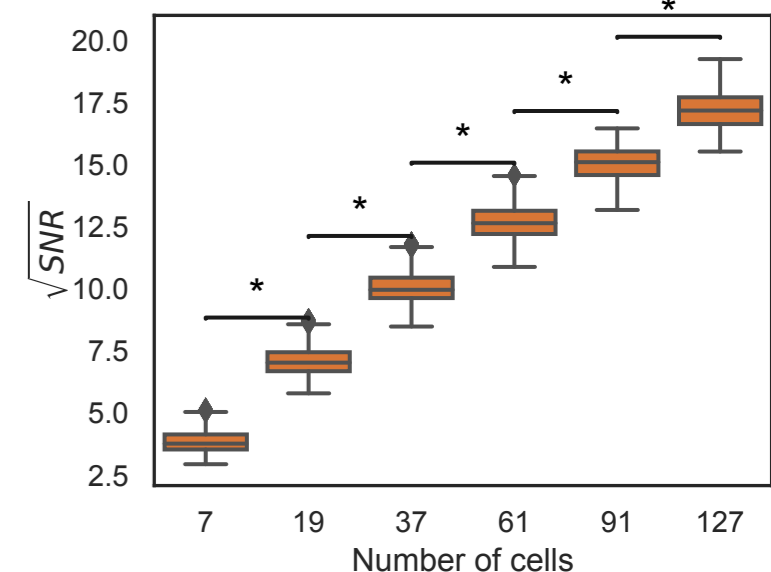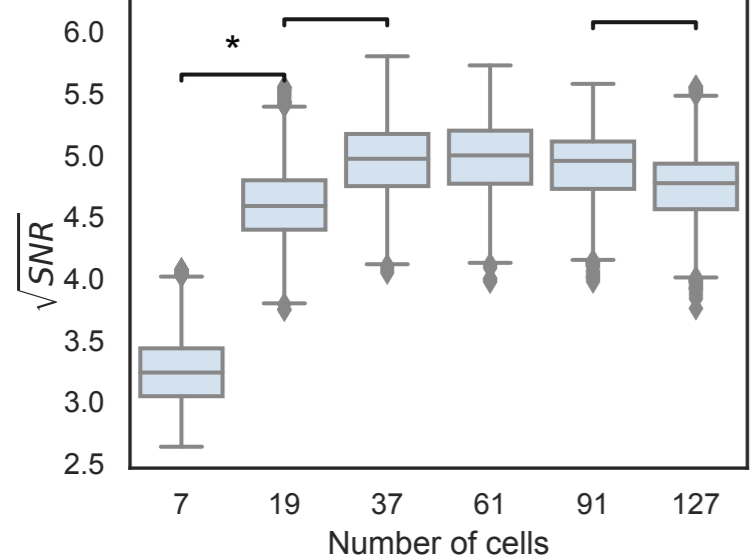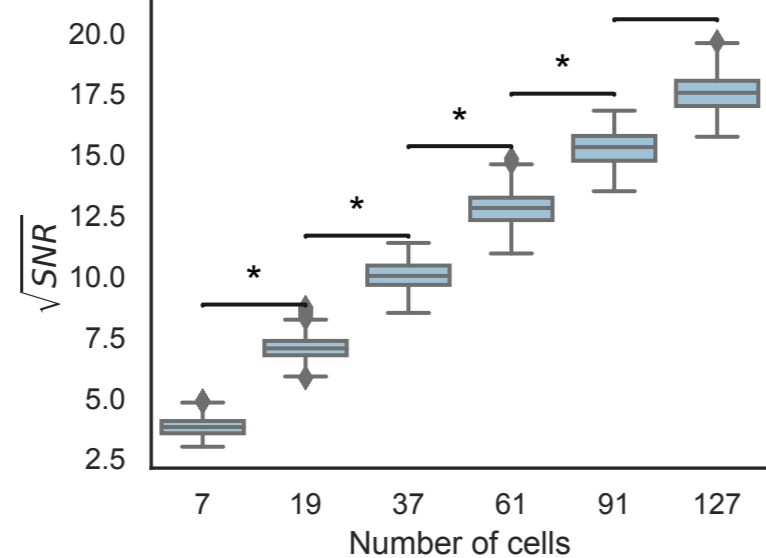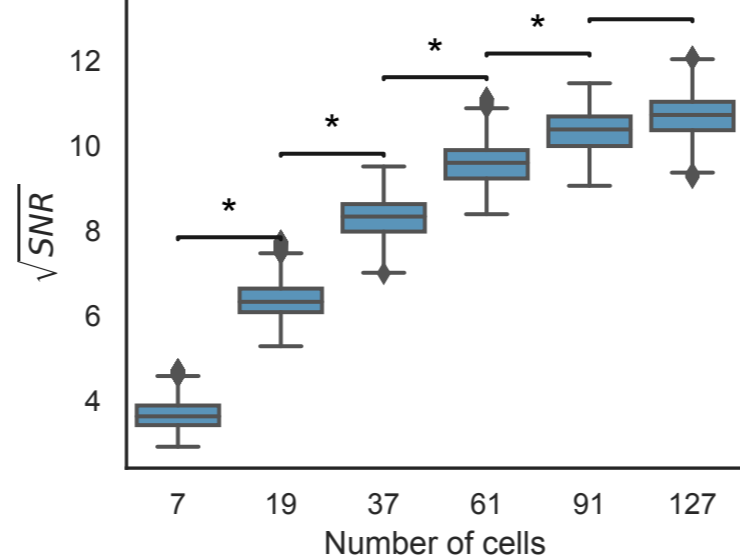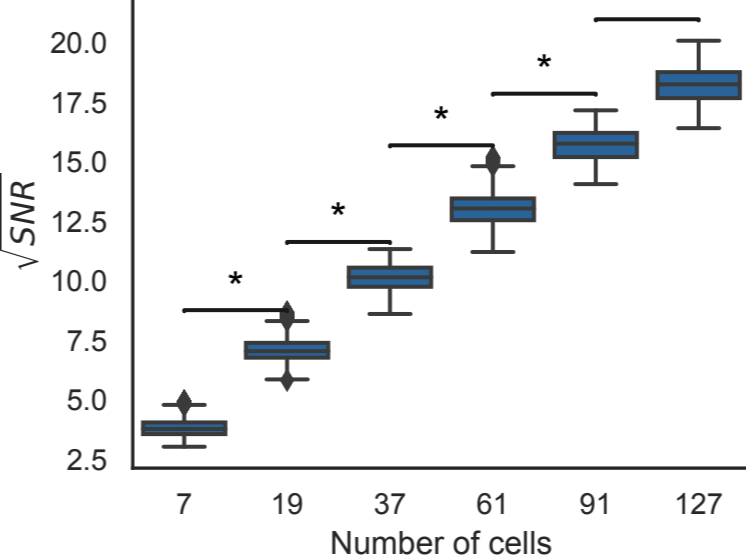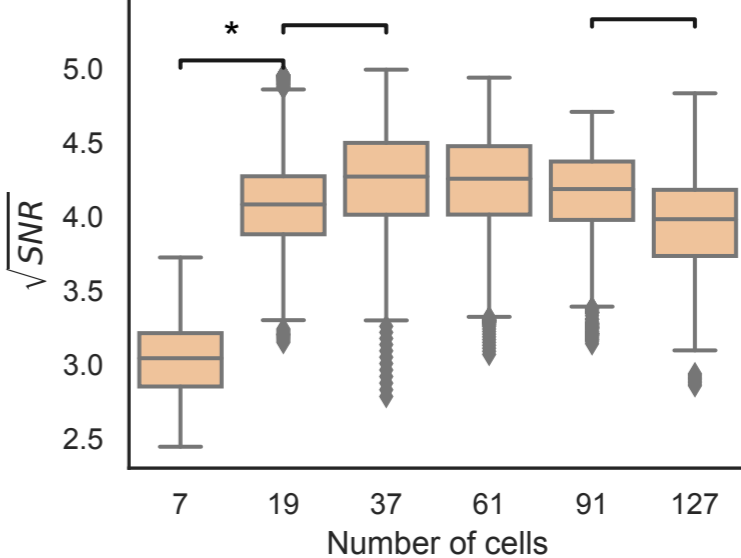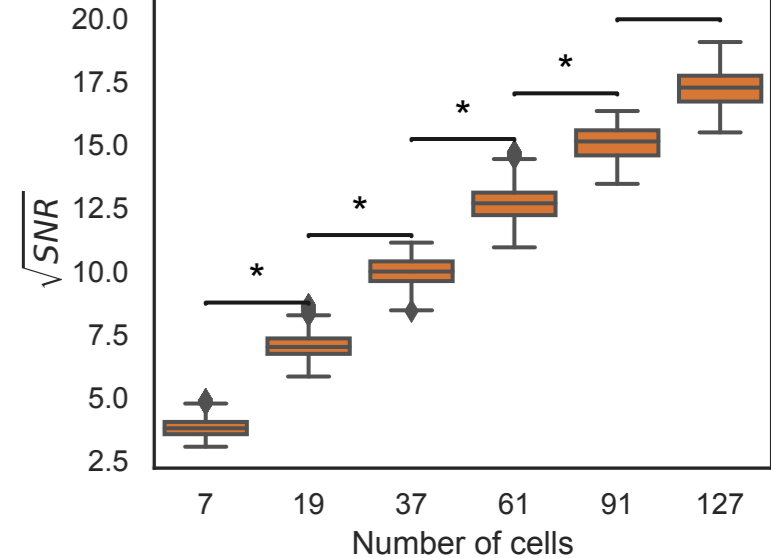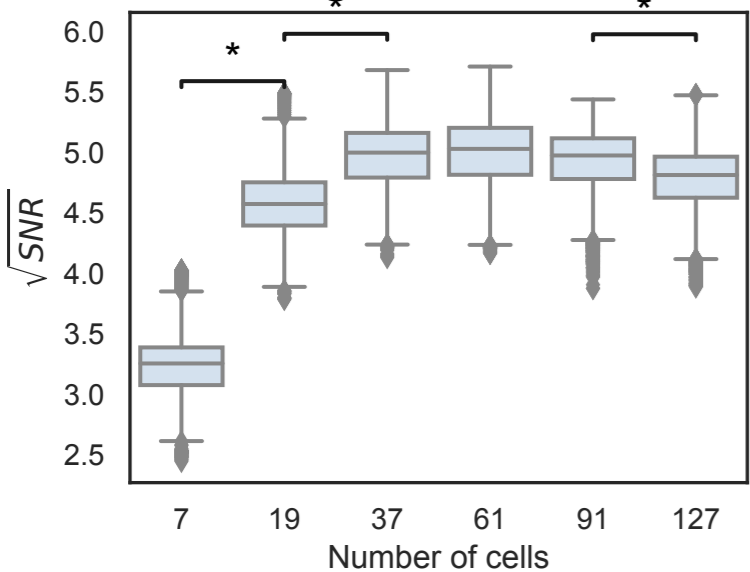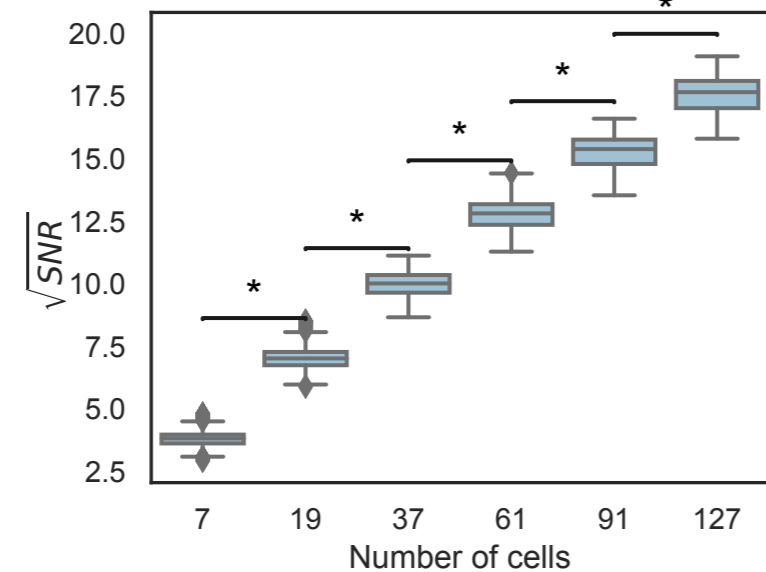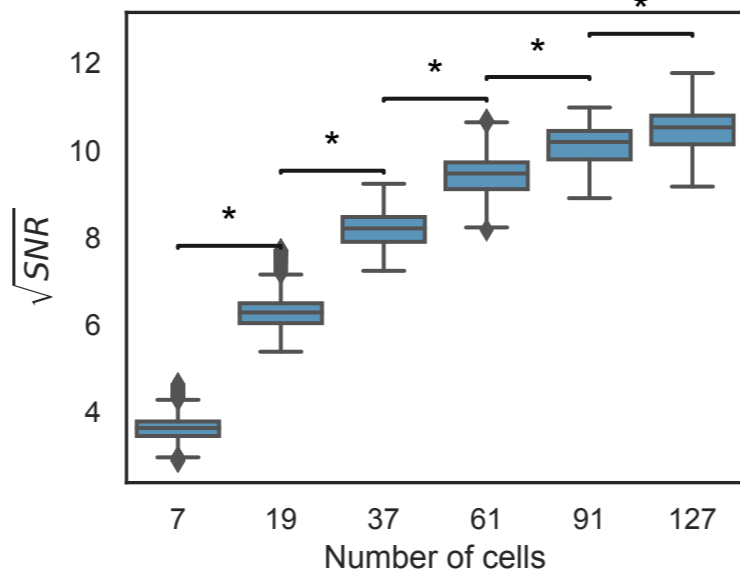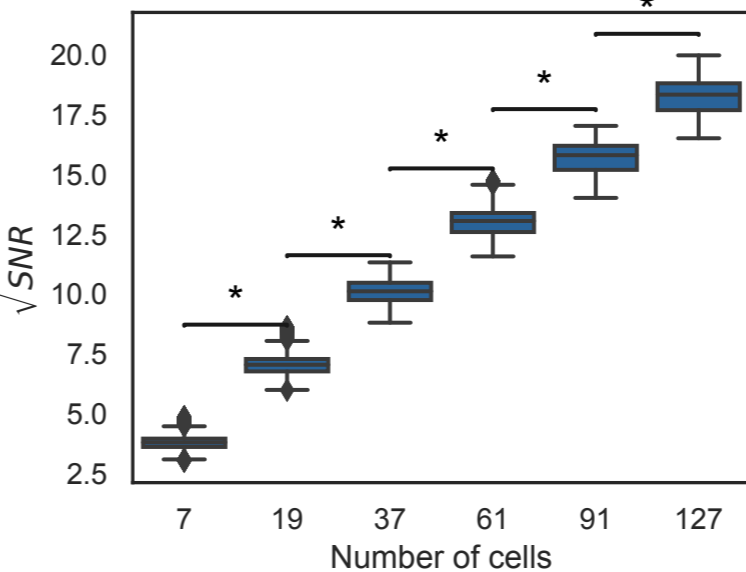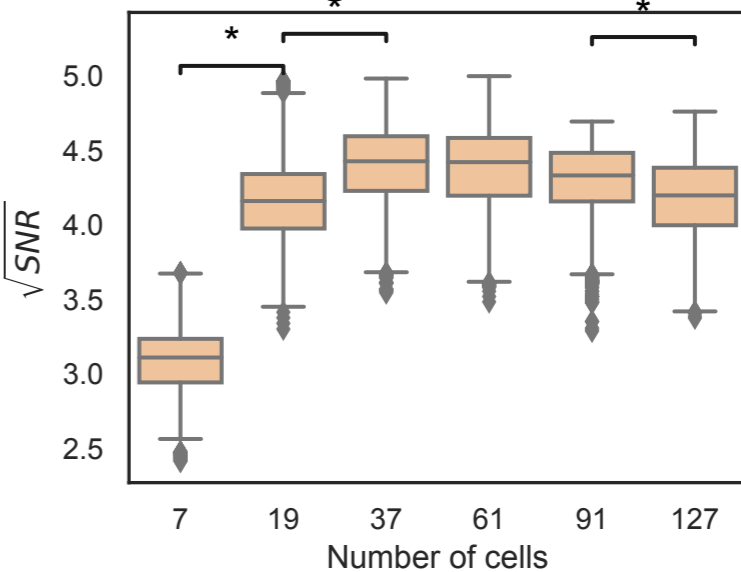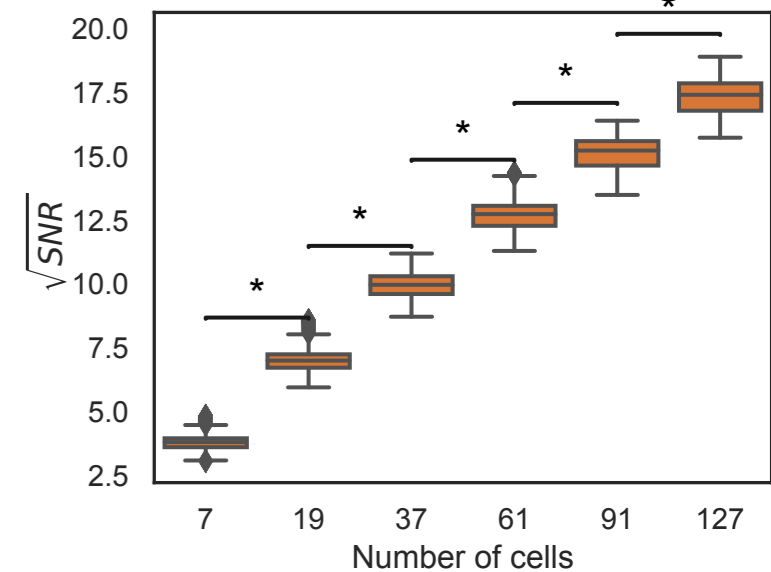

Supplement: S4 Fig — Box plots comparing the set of SNR values for configurations with different number of cells, for different values of the mean polygon number (rows). The results are obtained combining 10 sets of cell configurations. Weak-local (ISD) communication: D = 10.0 μm2/s, α = 1.0 s−1. Strong-local (ISD) communication: D = 10.0 μm2/s, α = 100.0 s−1. Weak-global (ISD) communication: D = 1000.0 μm2/s, α = 1.0 s−1. Strong-global (ISD) communication: D = 1000.0 μm2/s, α = 100.0 s−1. Weak (NNE) communication: γNNE = 0.1 s−1. Strong (NNE) communication: γNNE = 10.0 s−1. The ⋆ indicates statistically significant results (see Materials and methods for further details). (PDF) [file pcbi.1009552.s005.pdf]

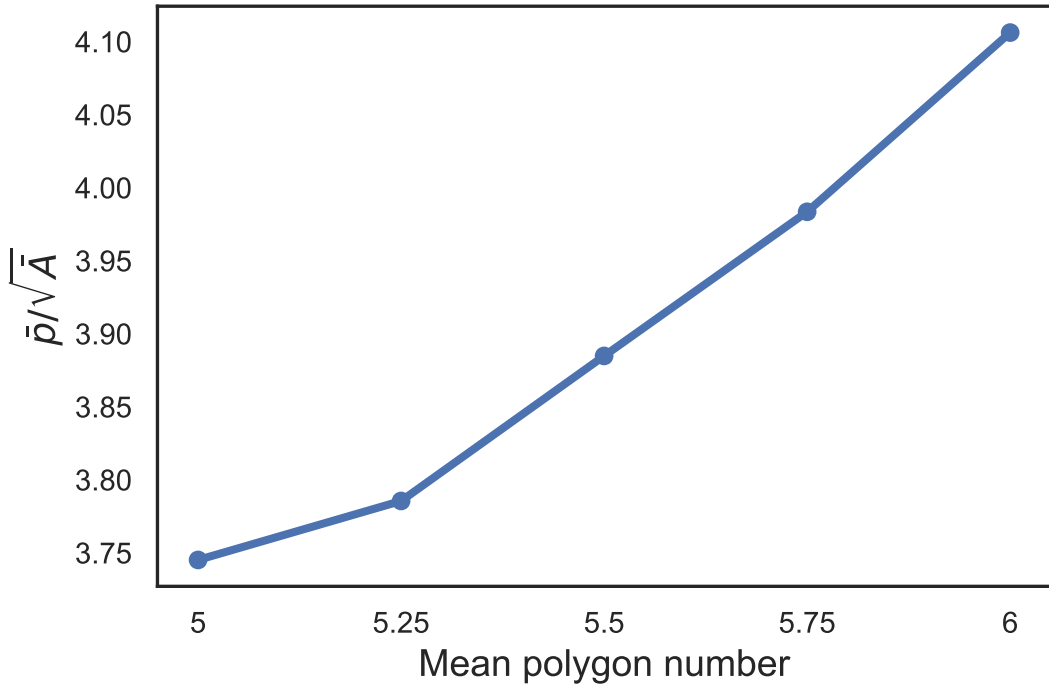

Supplement: S5 Fig — Mean of the ratio between perimeter and square root of the area as a function of the mean polygon number for the same cell configurations shown in Fig 6F, in the Weak-global (ISD) communication regime: D = 1000.0 μm2/s, α = 1.0 s−1. (PDF) [file pcbi.1009552.s006.pdf]

# ISD

# NNE

## Weak-local

## Strong-local

## Weak-global

## Strong-global

## Weak

## Strong

7

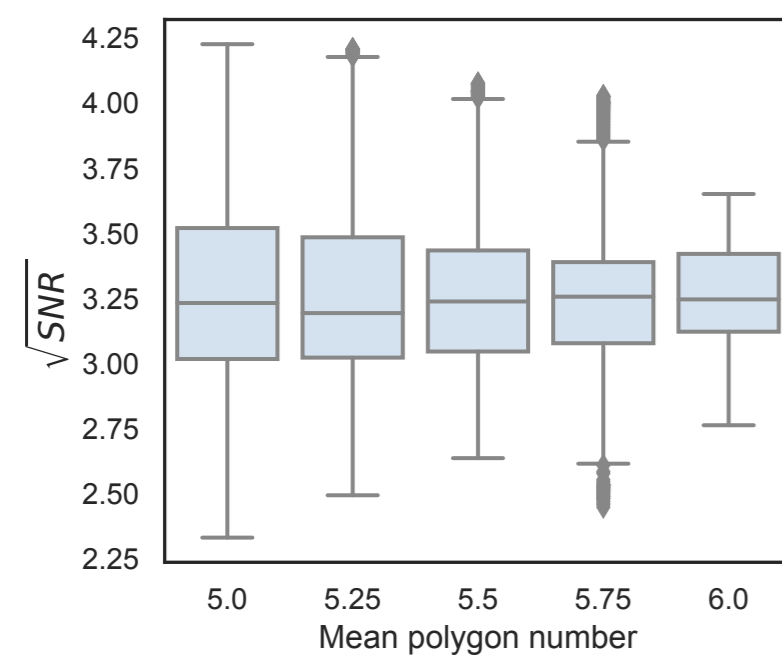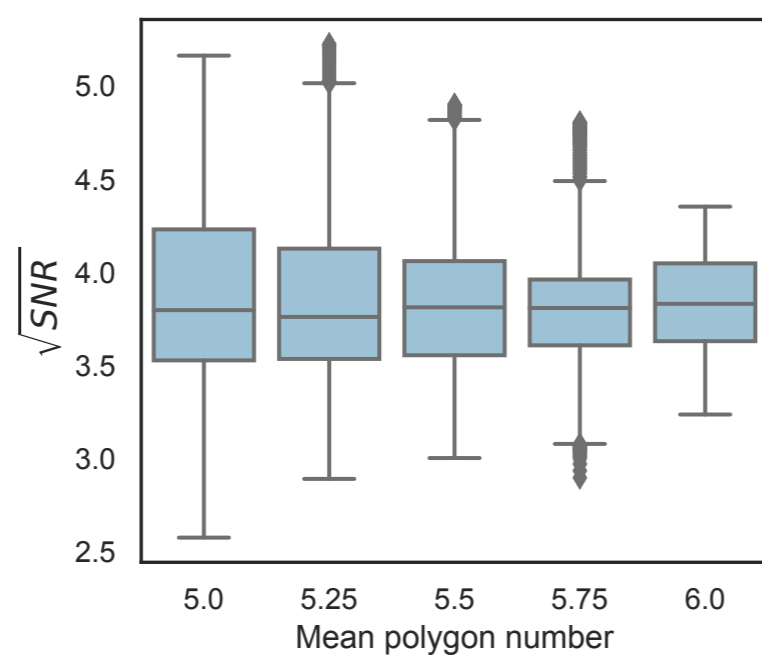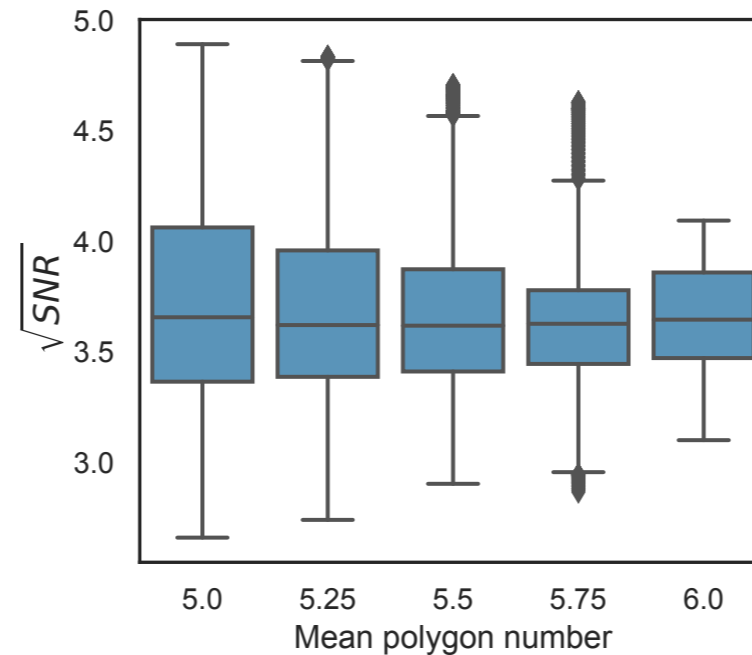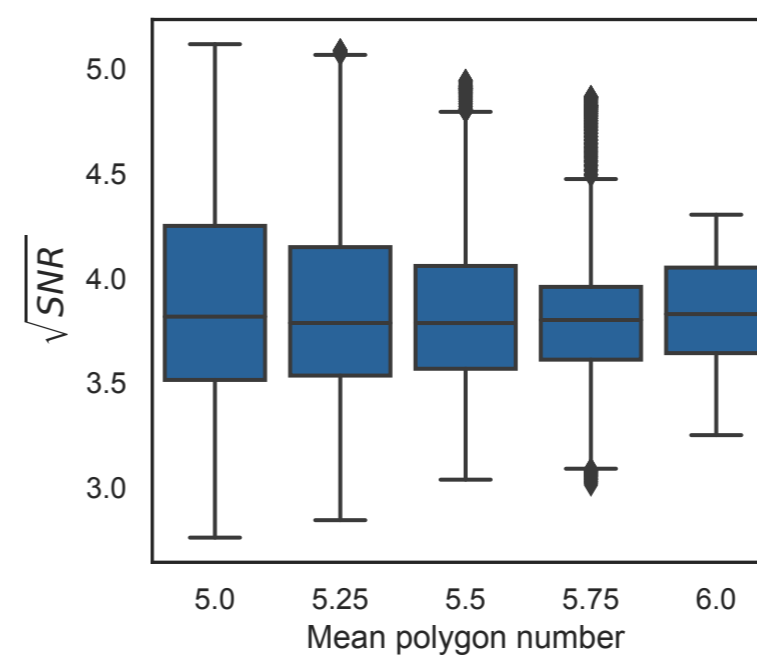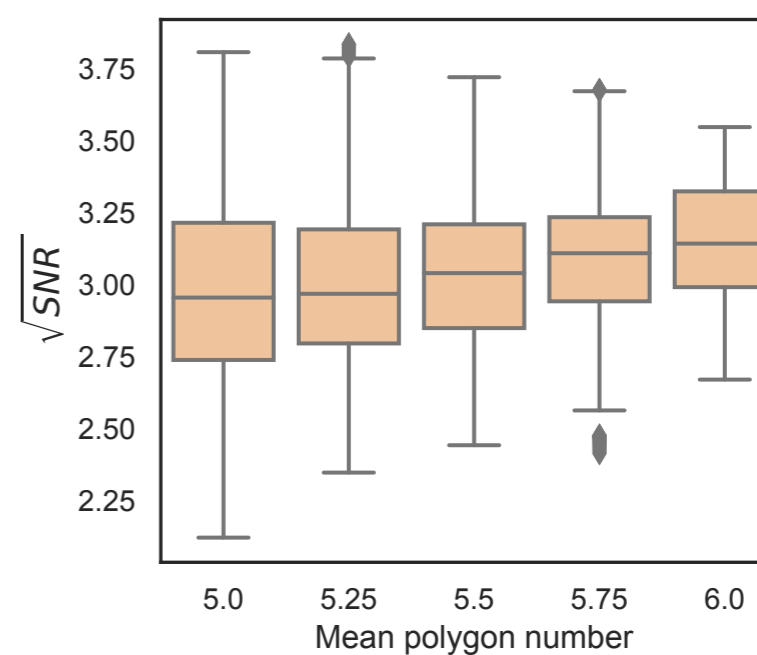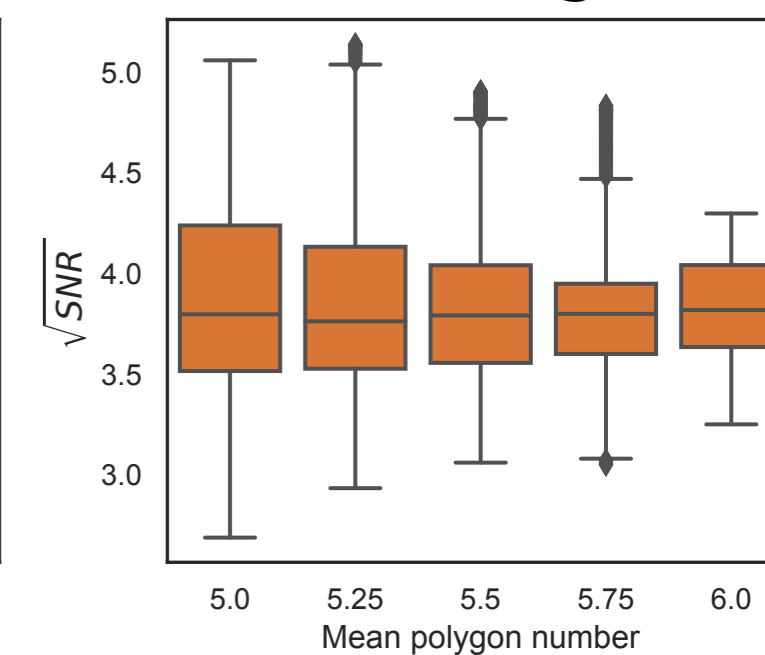

19

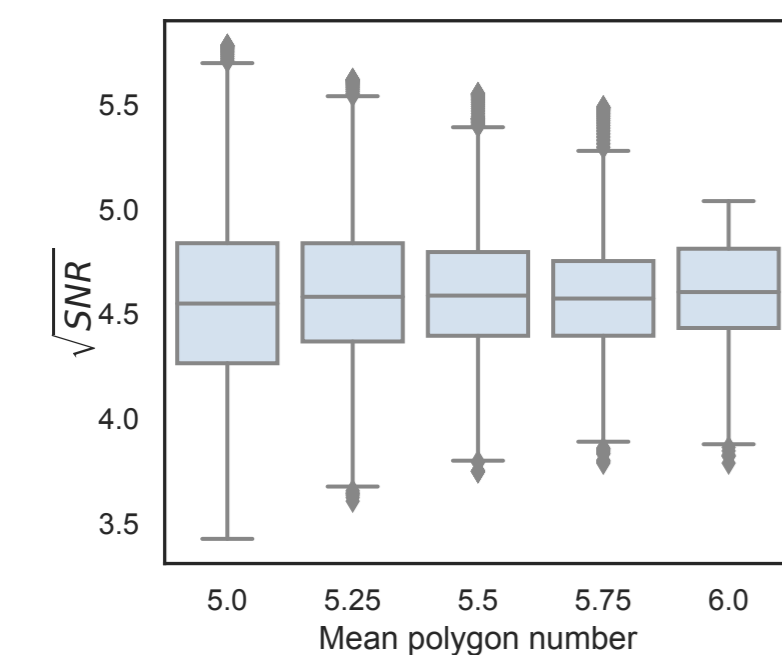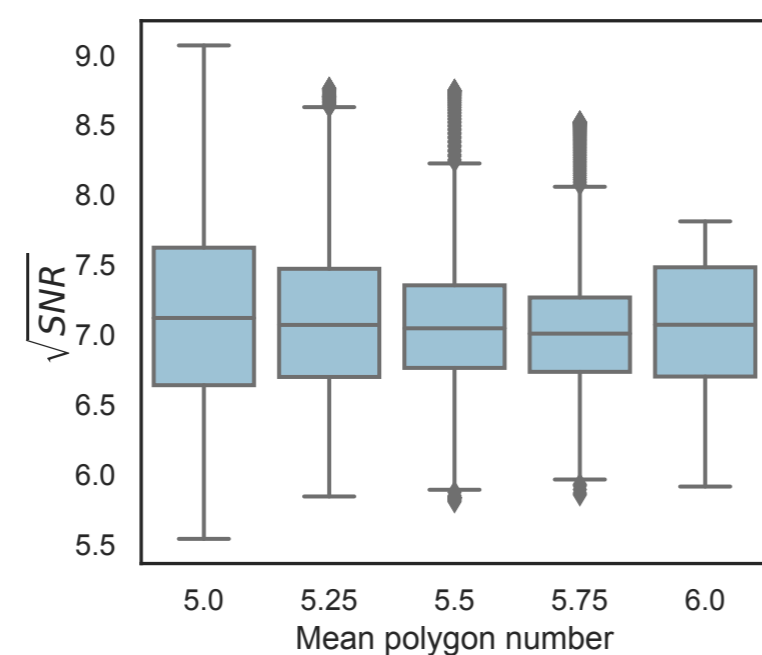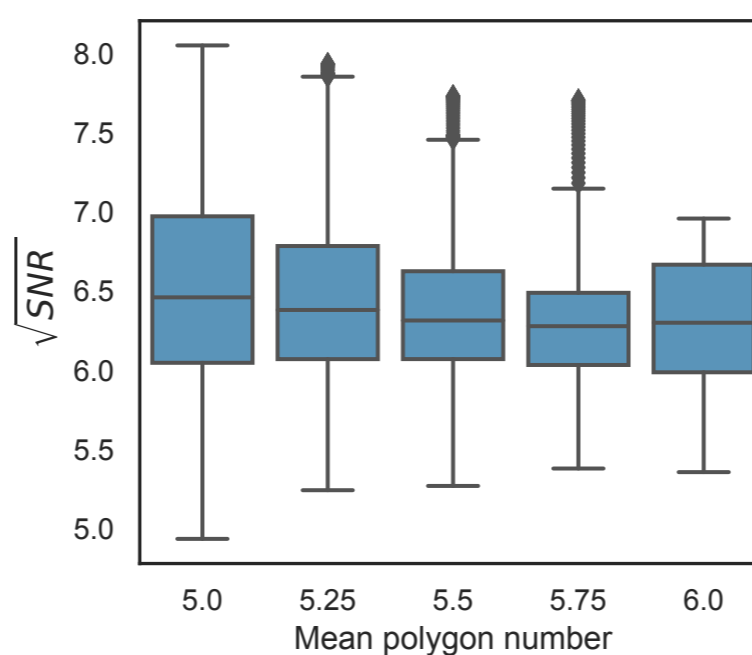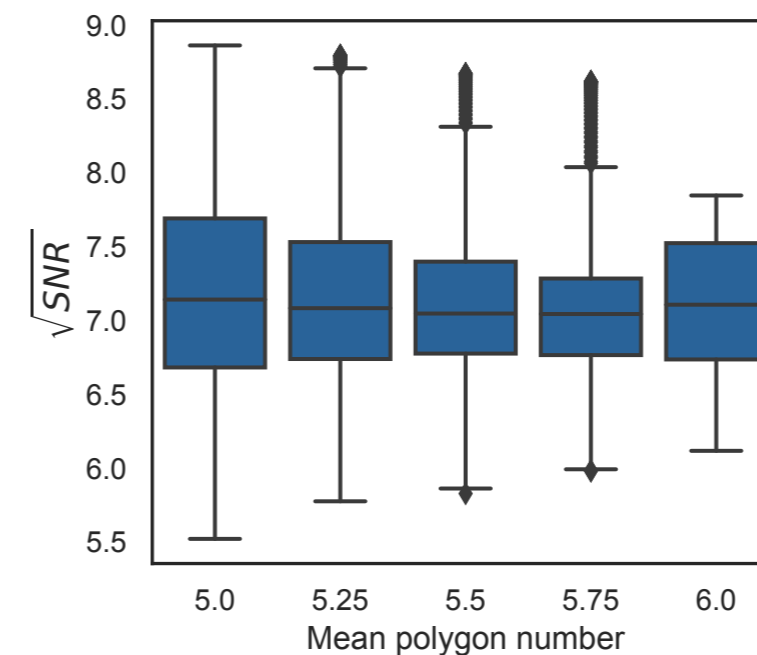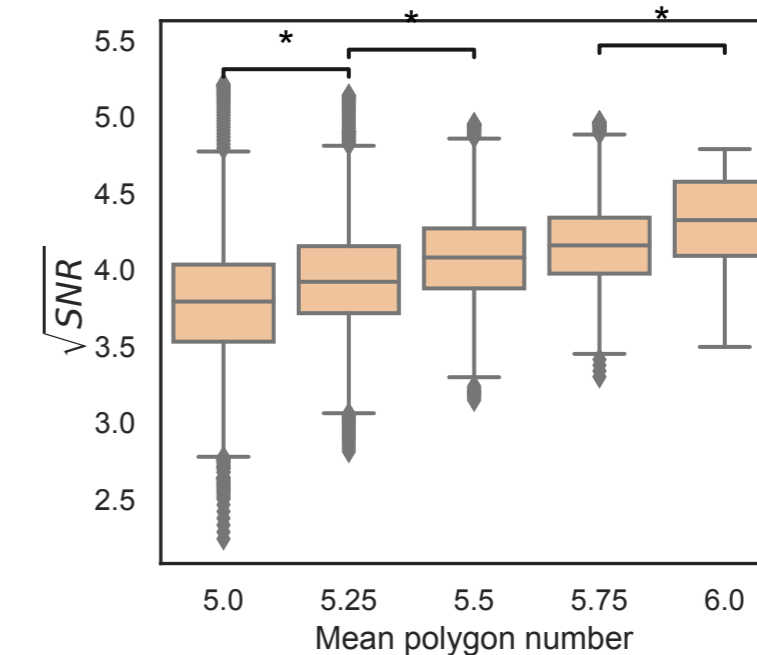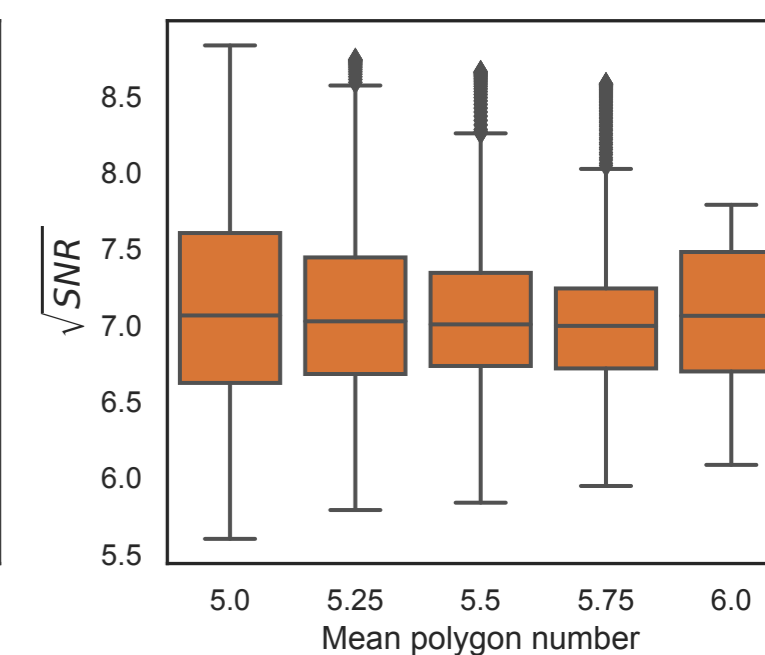

37

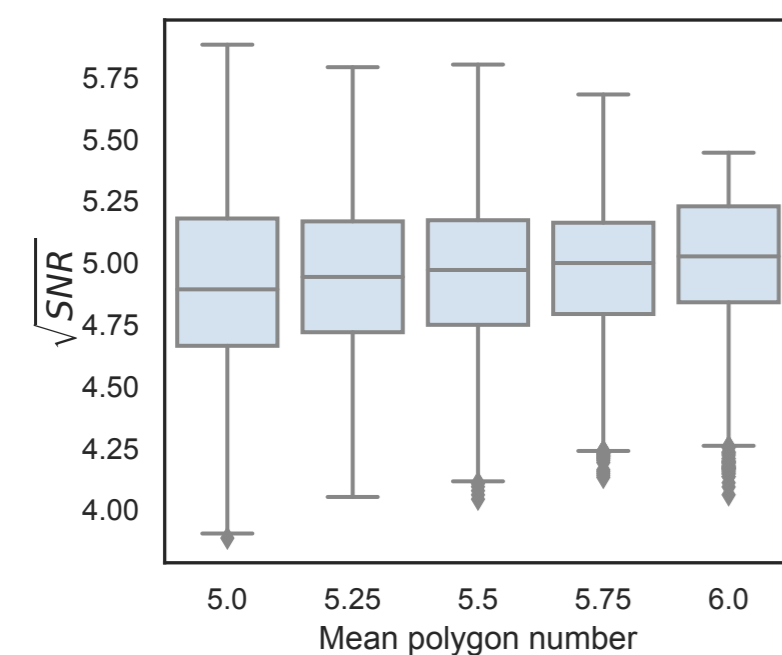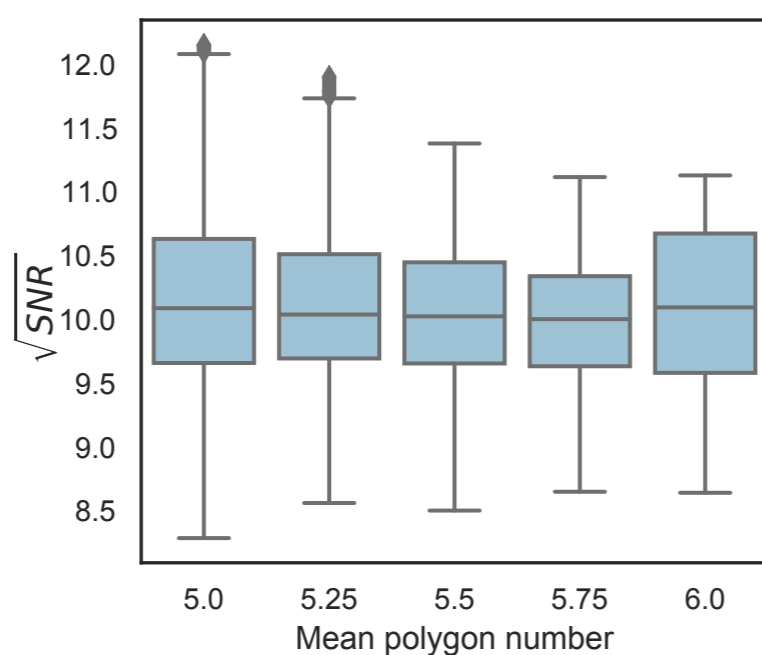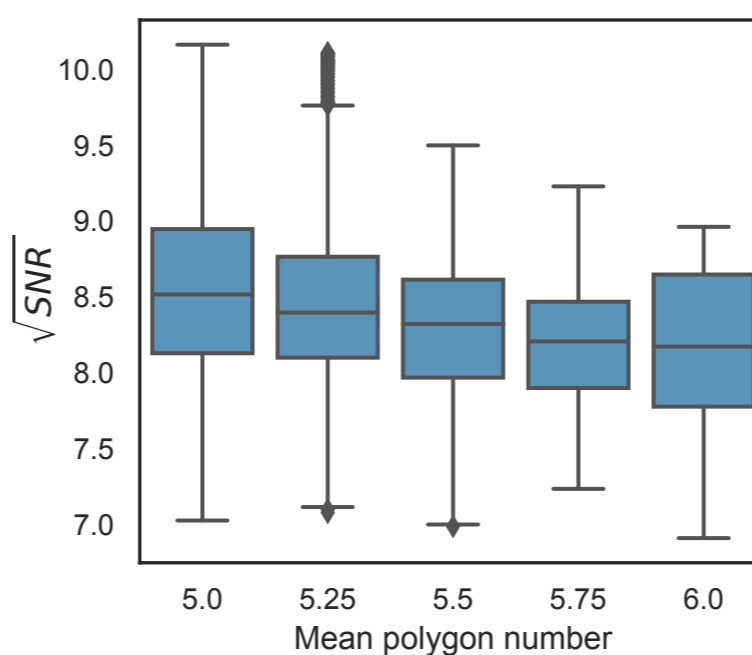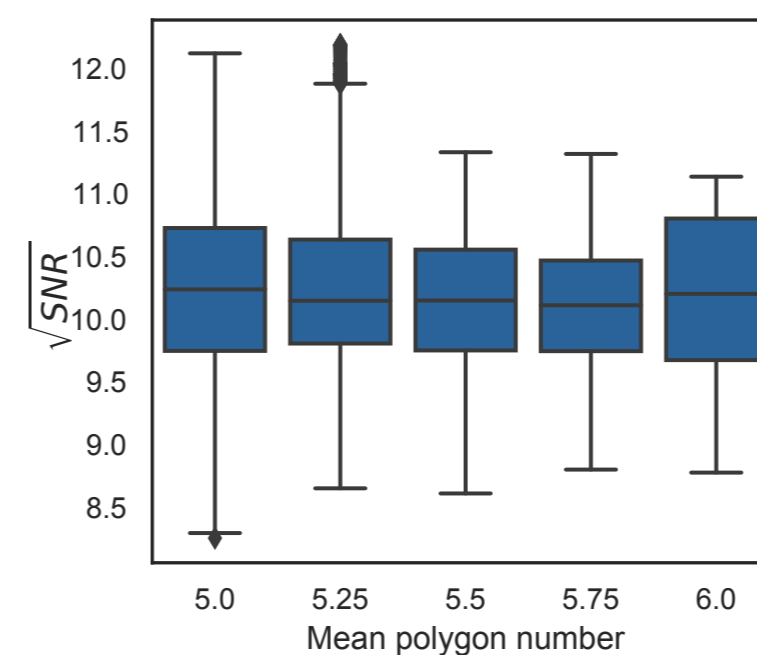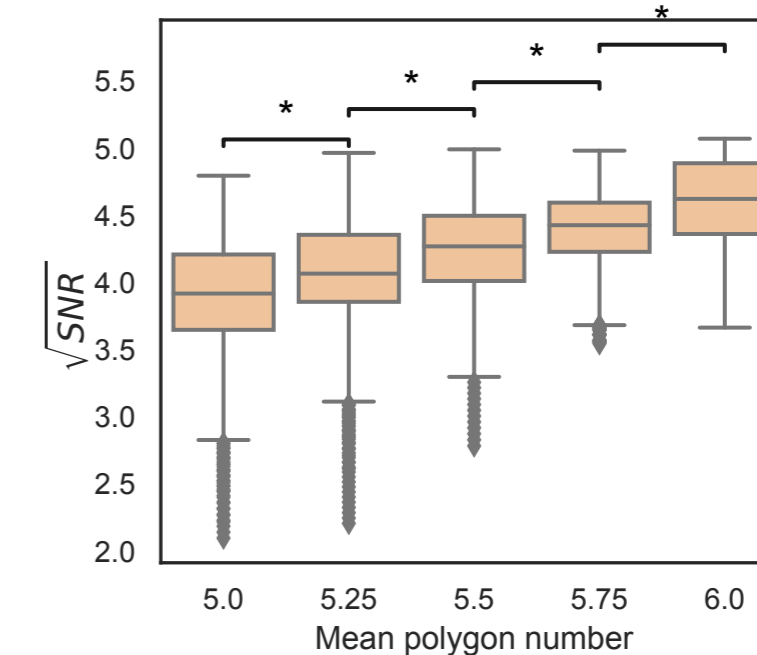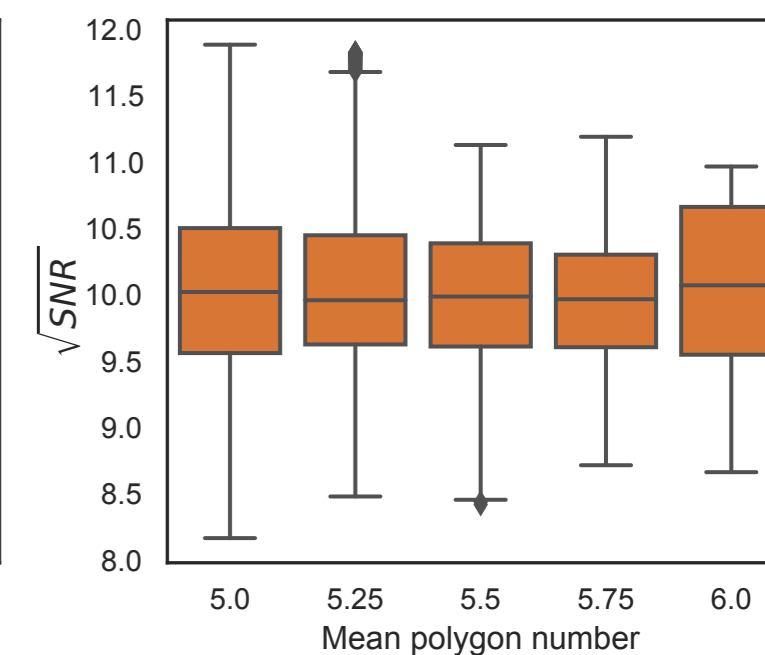

61

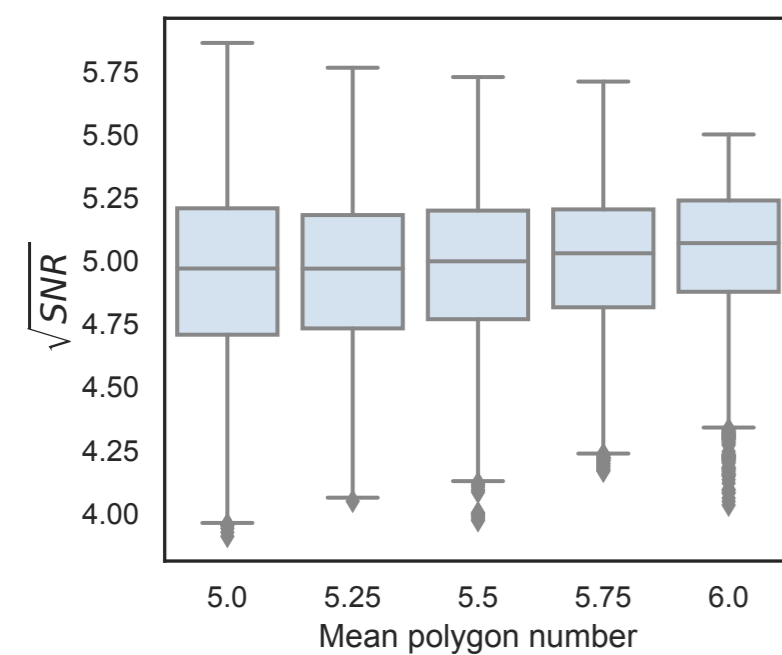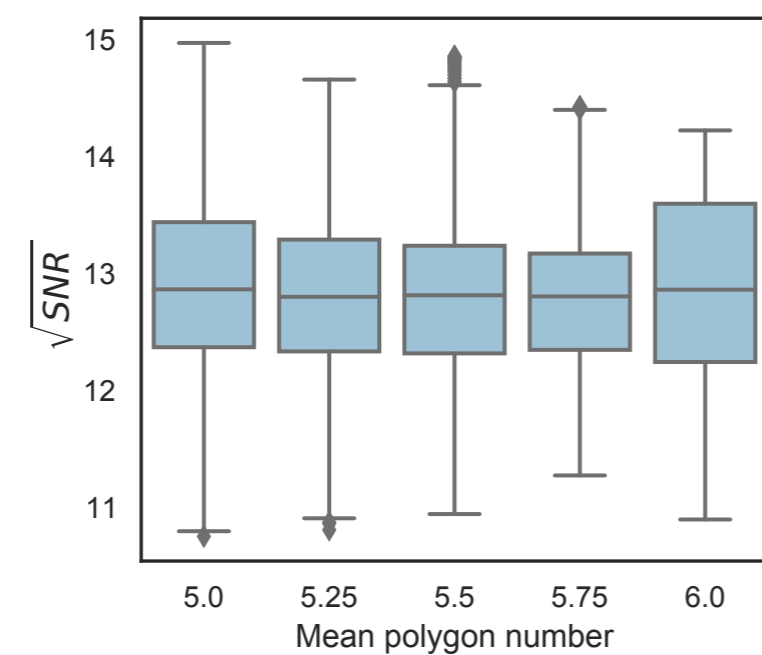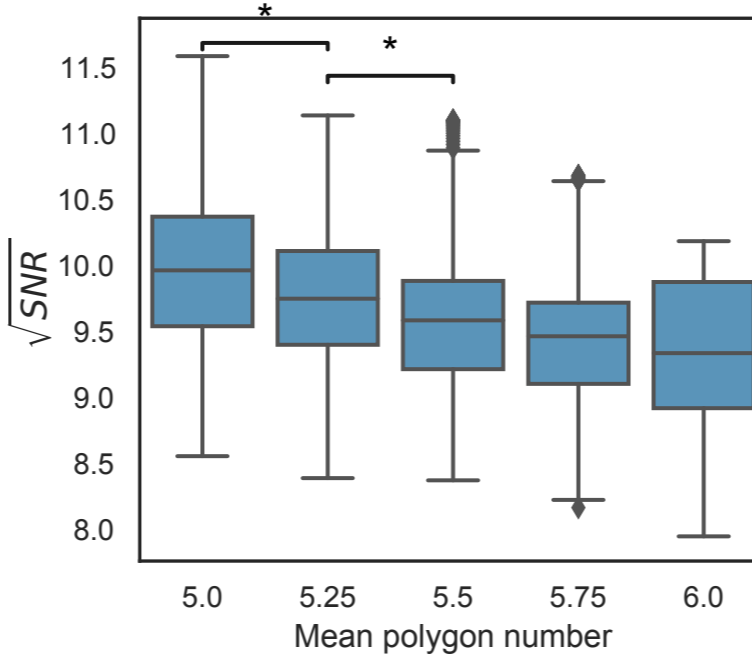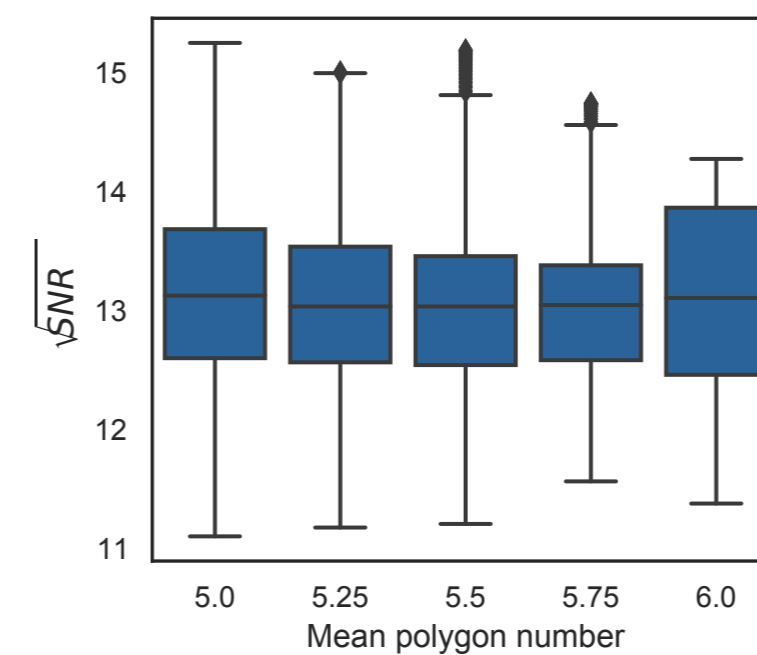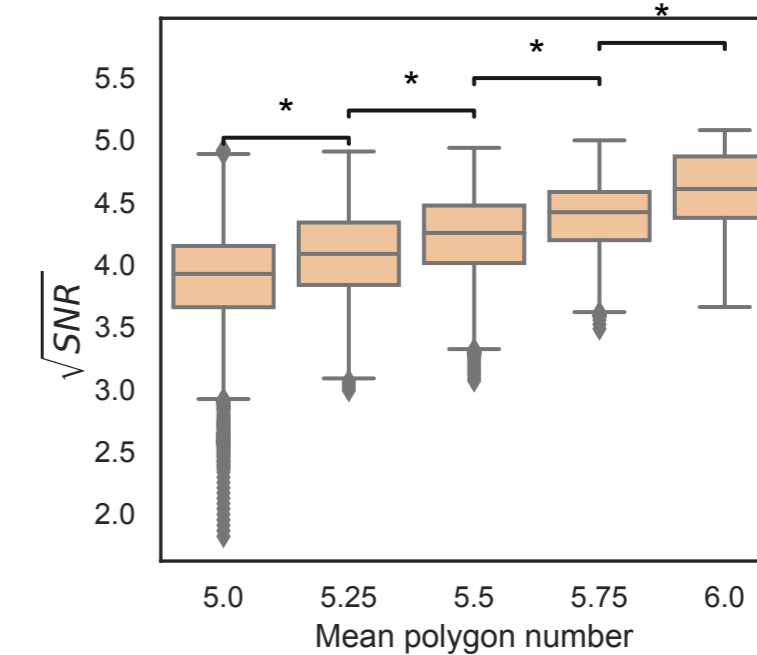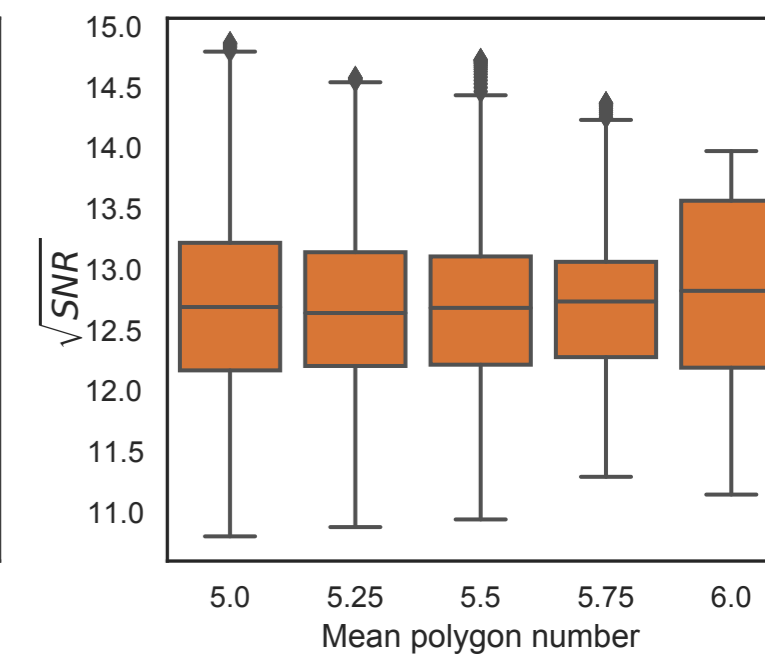

91

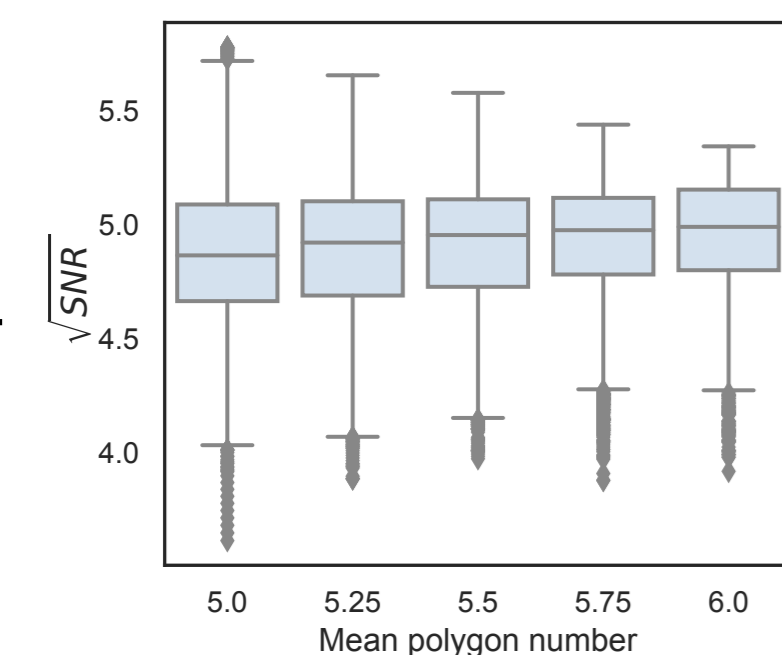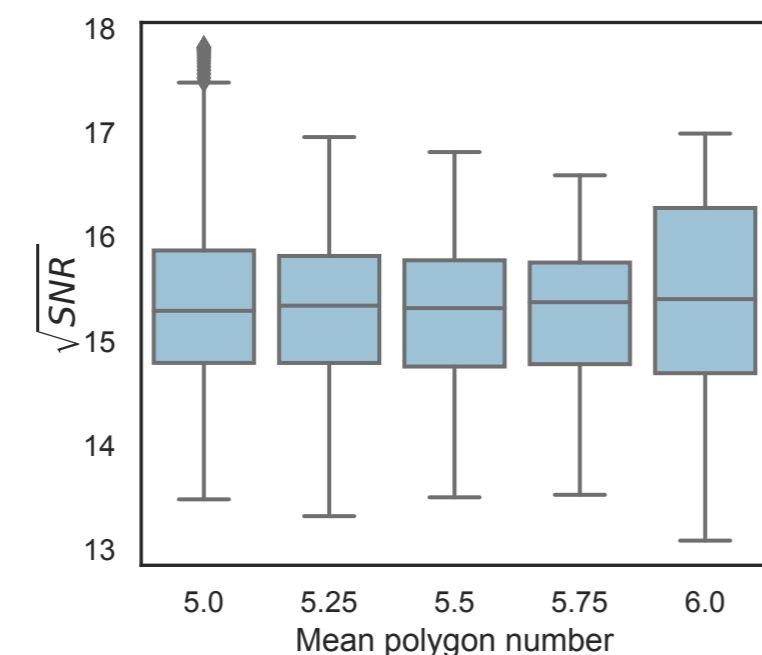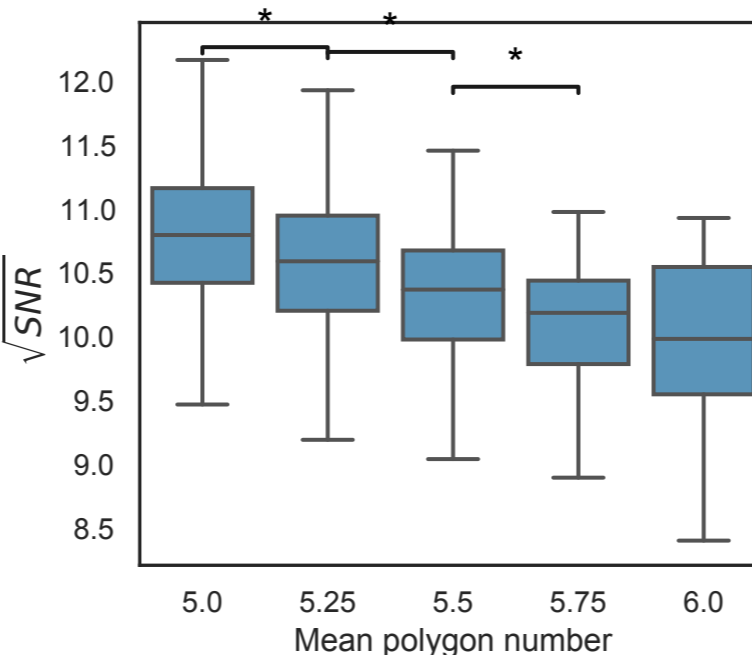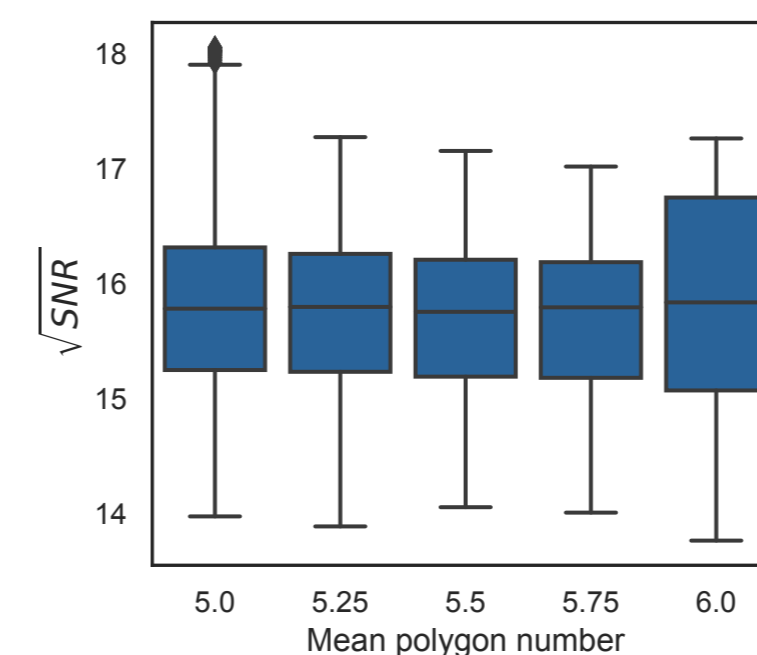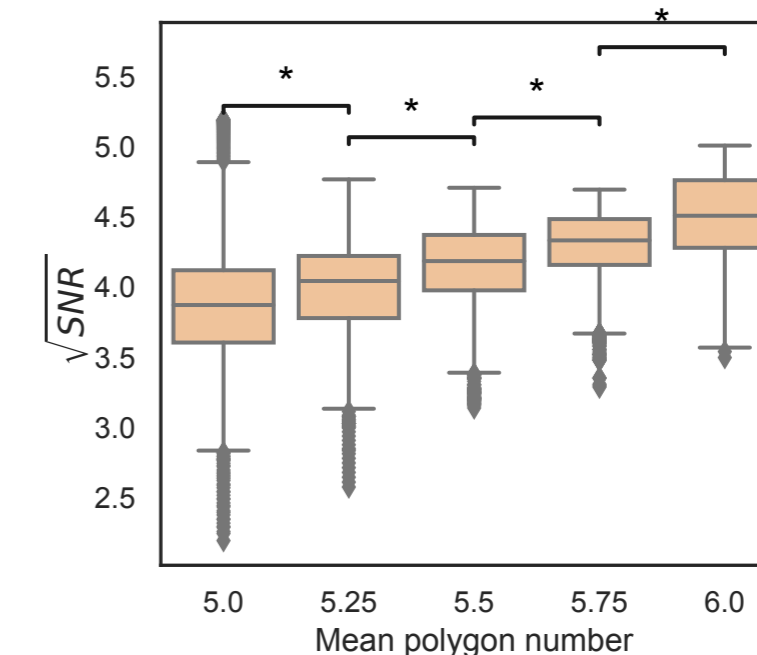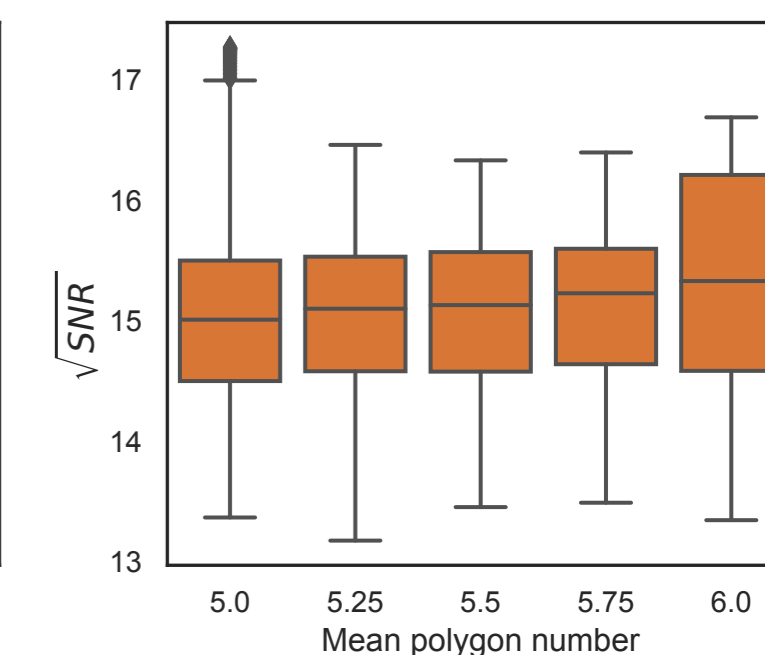

Supplement: S6 Fig — Box plots comparing the set of SNR values for configurations with different values of the mean polygon number, for different number of cells (rows). The results are obtained combining 10 sets of cell configurations. Weak-local (ISD) communication: D = 10.0 μm2/s, α = 1.0 s−1. Strong-local (ISD) communication: D = 10.0 μm2/s, α = 100.0 s−1. Weak-global (ISD) communication: D = 1000.0 μm2/s, α = 1.0 s−1. Strong-global (ISD) communication: D = 1000.0 μm2/s, α = 100.0 s−1. Weak (NNE) communication: γNNE = 0.1 s−1. Strong (NNE) communication: γNNE = 10.0 s−1. The ⋆ indicates statistically significant results (see Materials and methods for further details). (PDF) [file pcbi.1009552.s007.pdf]

7

Weak-local

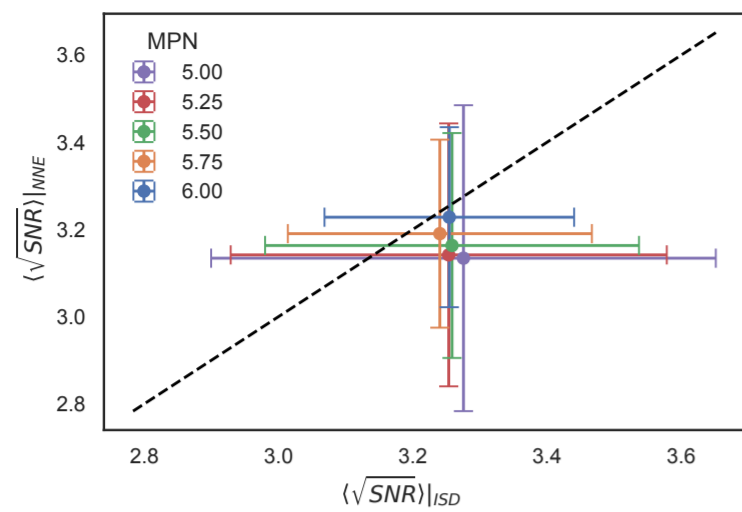

Strong-local

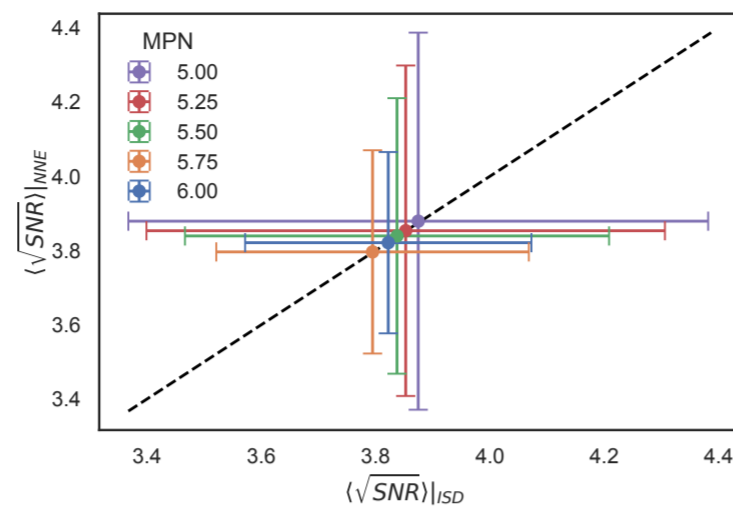

Weak-global

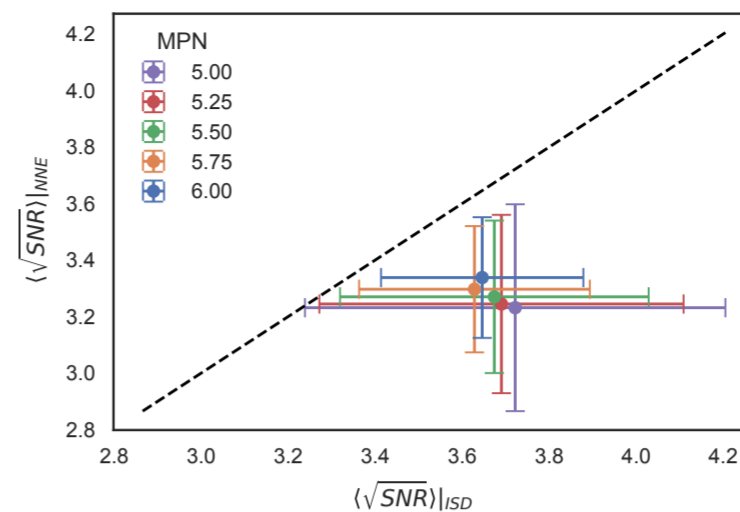

Strong-global

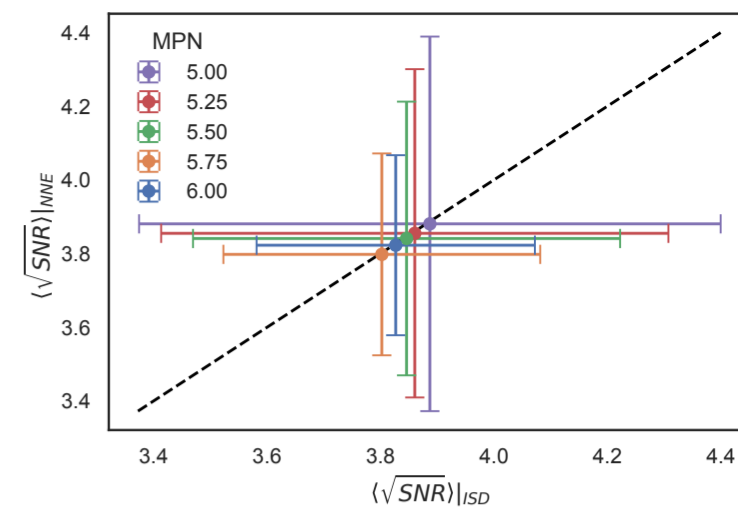

19

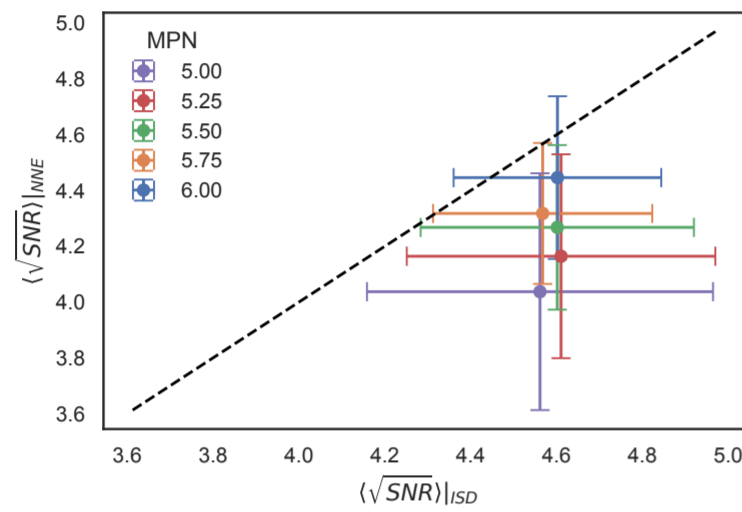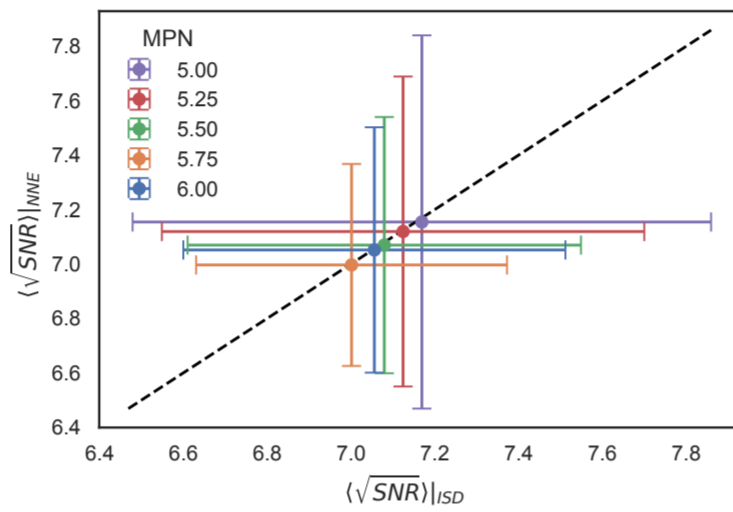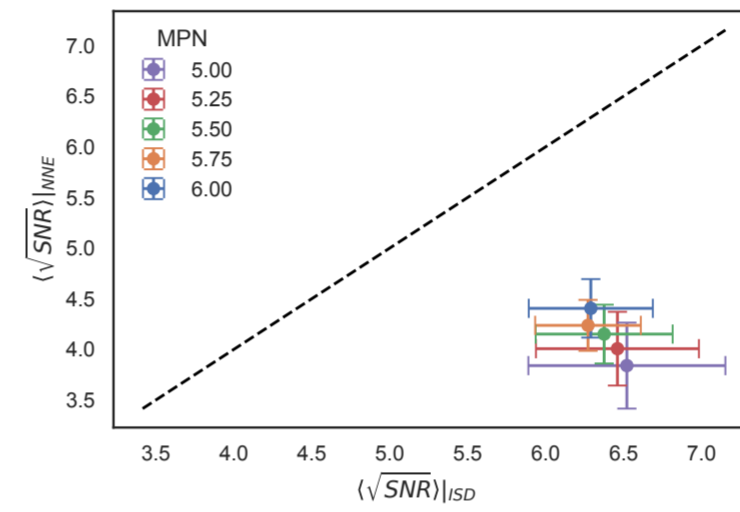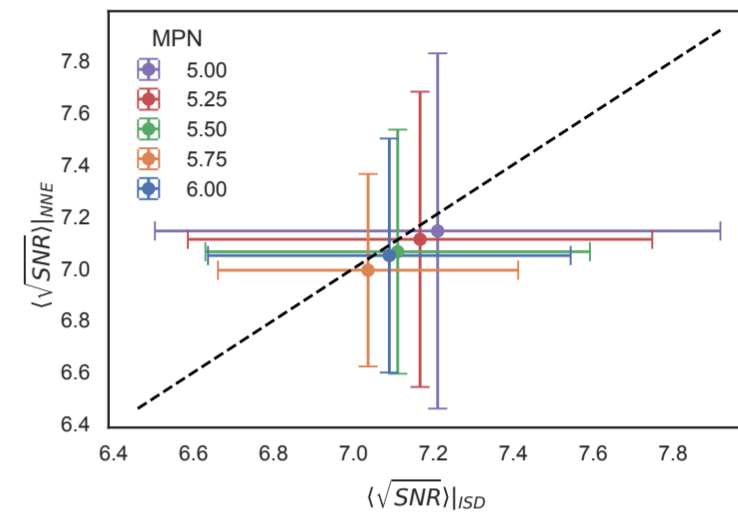

37

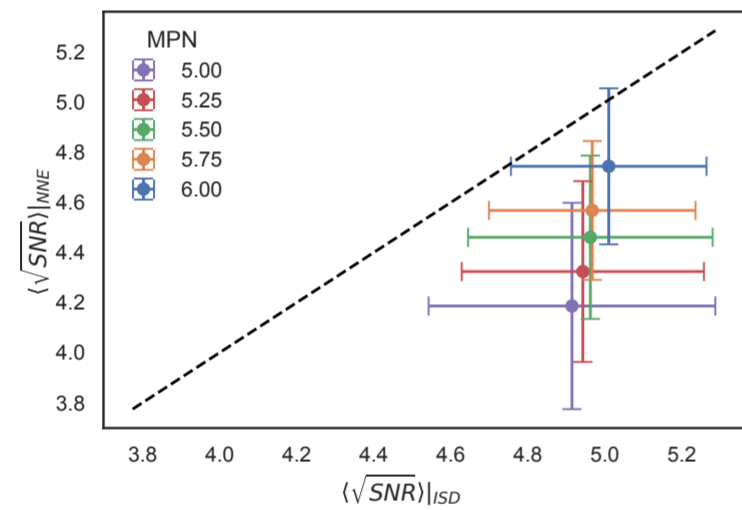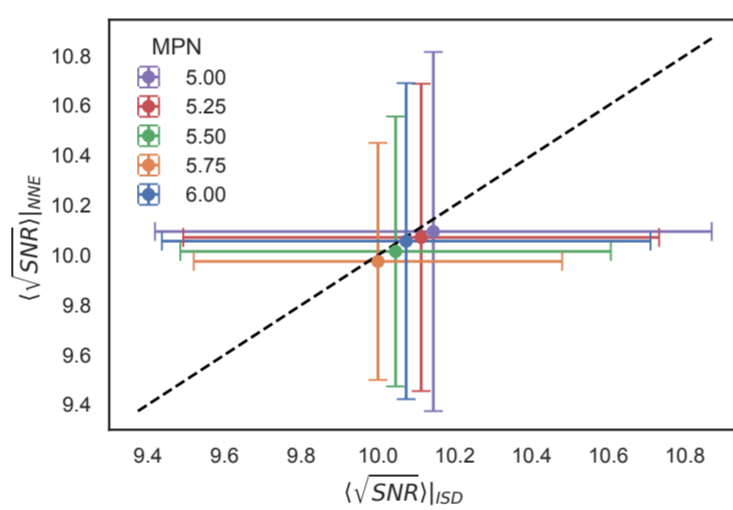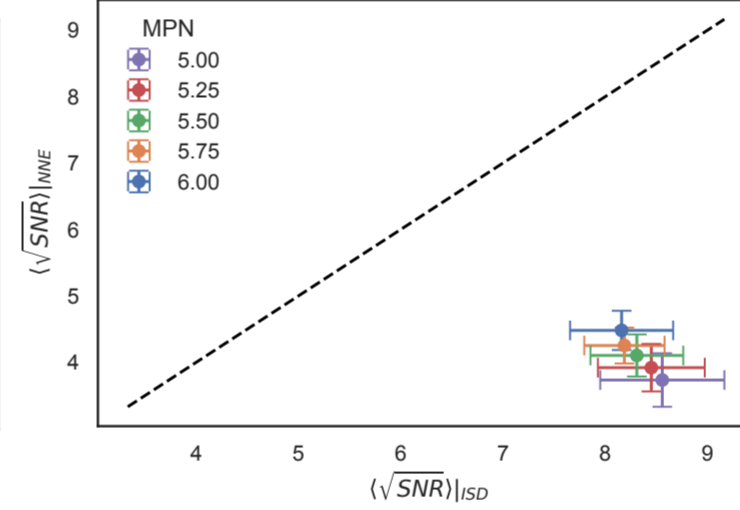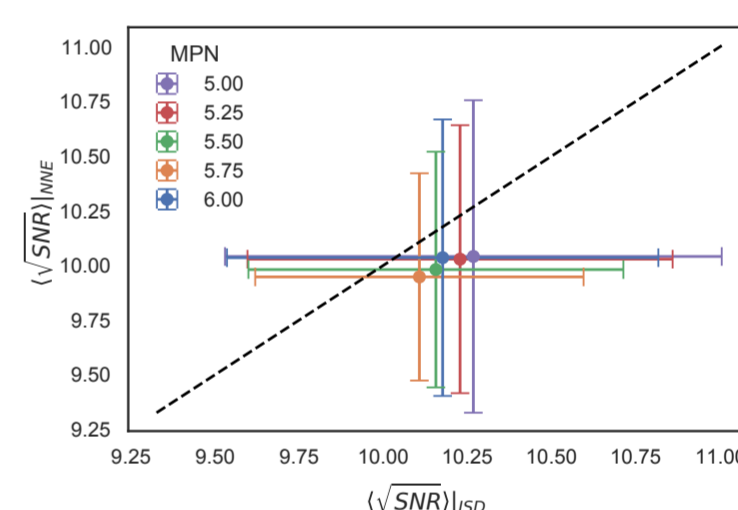

61

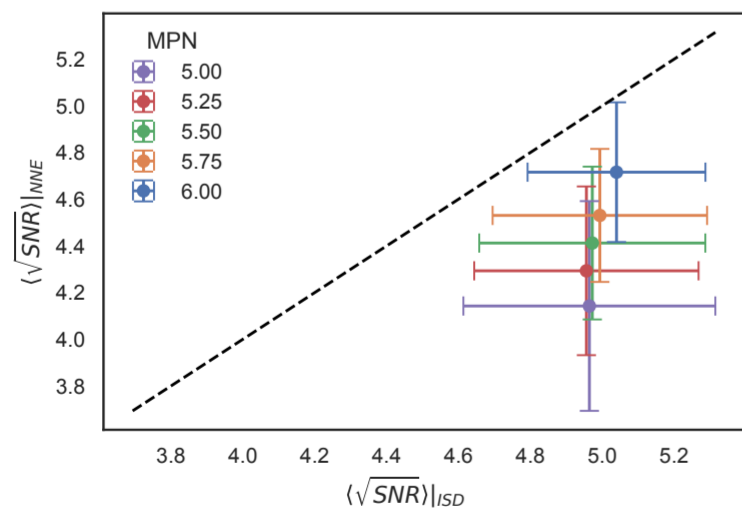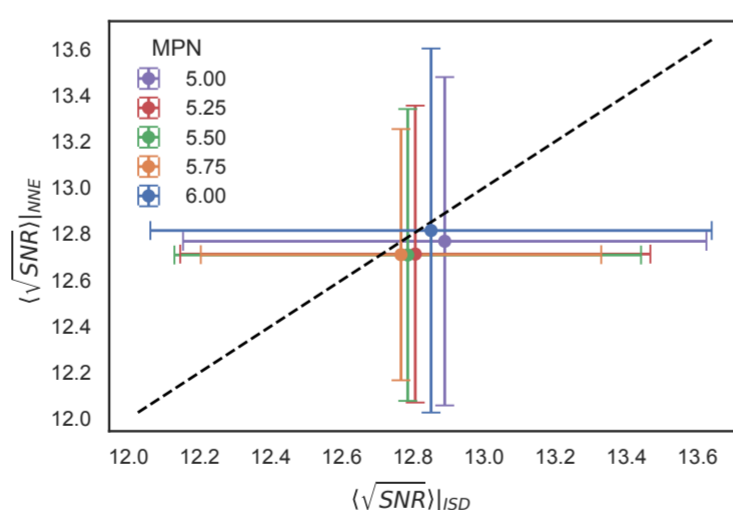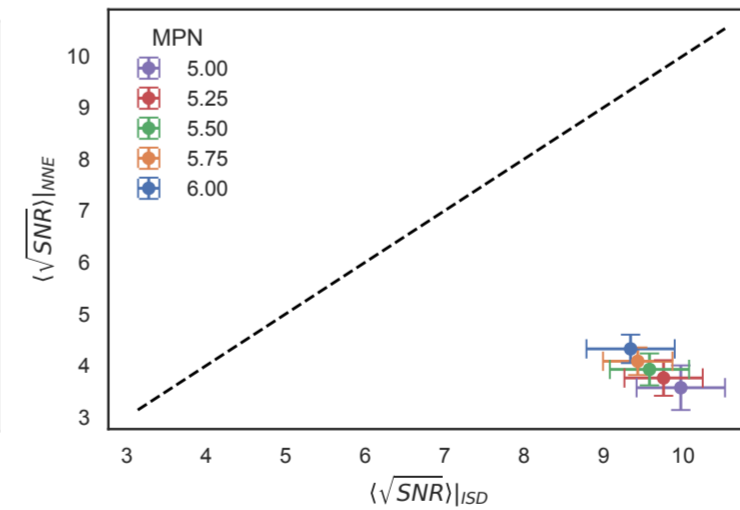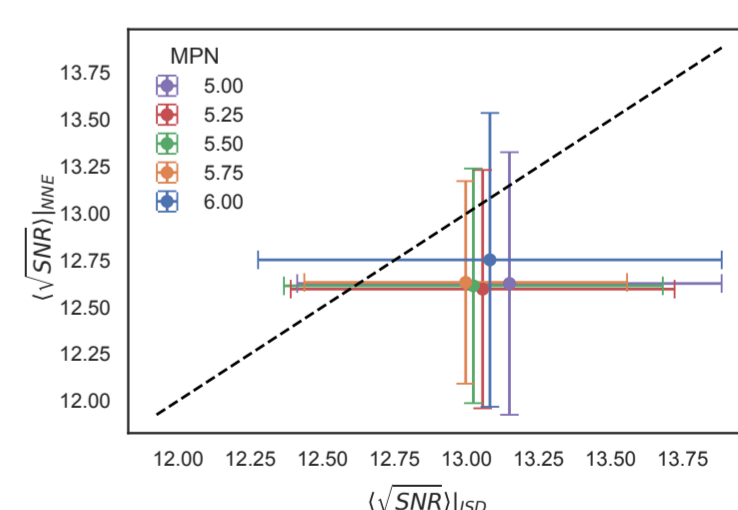

91

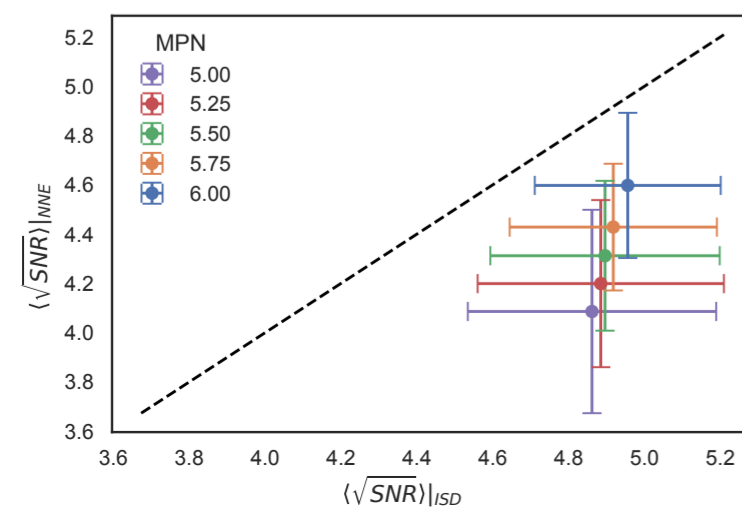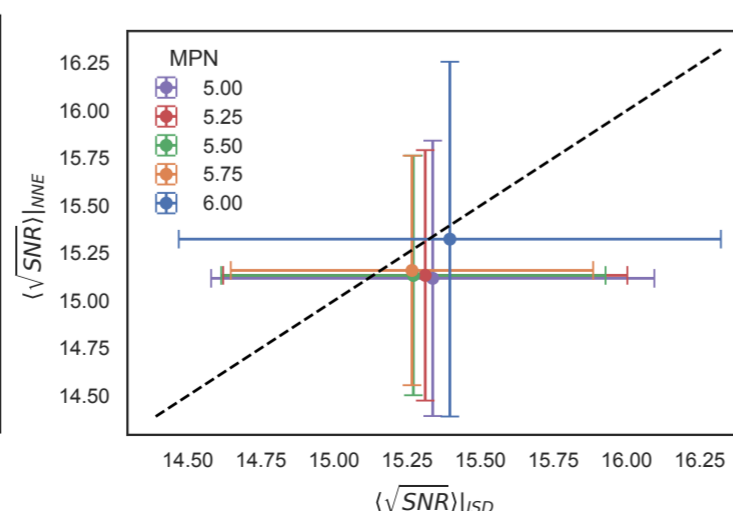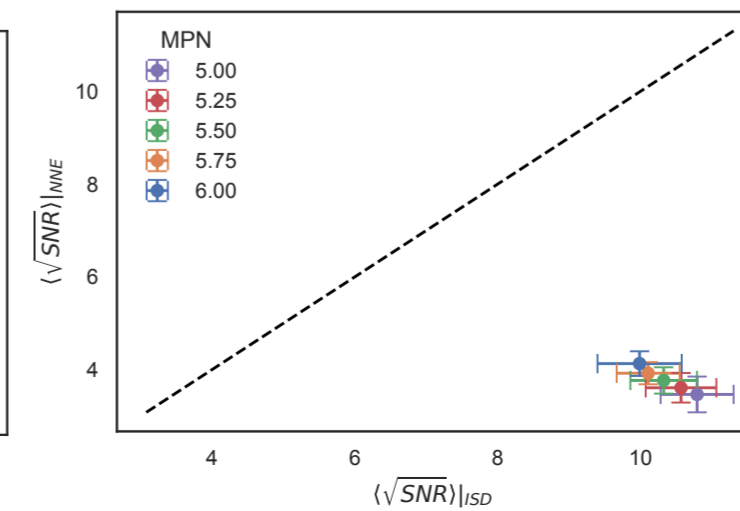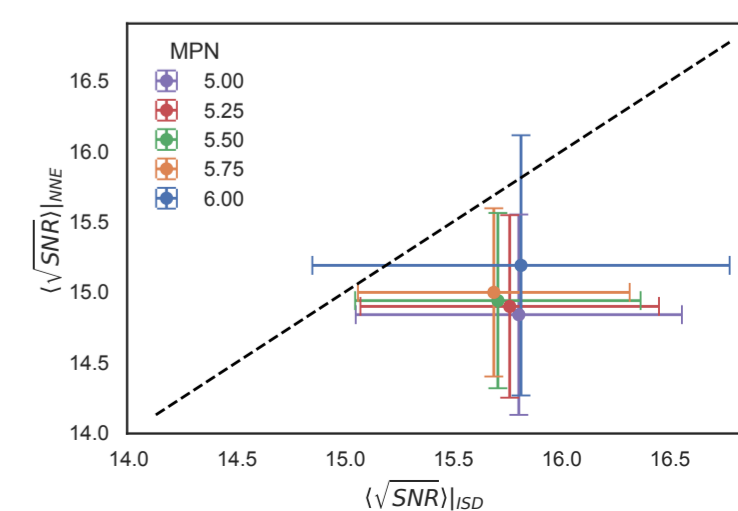

Supplement: S7 Fig — Scatter plots of the mean of the square root SNR computed on the edge cells for 10 sets of configurations, for the ISD (x-axis) and NNE (y-axis) communication modes. In each plot we present the results for different values of the mean polygon number (MPN); results for different number of cells are shown in the rows. The dashed black line indicates the bisector. Error bars are given by the standard deviation. Weak-local communication: D = 10.0 μm2/s, α = 1.0 s−1. Strong-local communication: D = 10.0 μm2/s, α = 100.0 s−1. Weak-global communication: D = 1000.0 μm2/s, α = 1.0 s−1. Strong-global communication: D = 1000.0 μm2/s, α = 100.0 s−1. (PDF) [file pcbi.1009552.s008.pdf]
