# Supplementary material for: The role of cell geometry and cell-cell communication in gradient sensing
Source: PLoS Comput Biol. 2022 Mar 14;18(3):e1009552. doi: 10.1371/journal.pcbi.1009552 (PMC8963572; doi:10.1371/journal.pcbi.1009552)
Supplement: S1 Table — We report the values of the CLES−0.5, which vary between −0.5 and 0.5 (see Materials and methods for details), for the comparisons between the distributions of the SNR for different number of cells, shown in Fig 5 and S4 Fig. A comparison is considered statistically significant if the p-value from a Wilcoxon rank sum test is smaller than 0.05 and |CLES − 0.5| > 0.1. NS indicates that the comparison is not significant, according to this criterion. (PDF) [file pcbi.1009552.s009.pdf]

# cells	ISD				NNE	
	Weak-local	Strong-local	Weak-global	Strong-global	Weak	Strong
7 - 19	-0.50	-0.50	-0.50	-0.50	-0.50	-0.50
19 - 37	-0.38	-0.50	-0.50	-0.50	-0.25	-0.50
37 - 61	NS	-0.50	-0.44	-0.50	NS	-0.50
61 - 91	NS	-0.49	-0.27	-0.49	0.11	-0.48
91 - 127	0.15	-0.45	-0.15	-0.46	0.12	-0.44
