# Supplementary material for: The role of cell geometry and cell-cell communication in gradient sensing
Source: PLoS Comput Biol. 2022 Mar 14;18(3):e1009552. doi: 10.1371/journal.pcbi.1009552 (PMC8963572; doi:10.1371/journal.pcbi.1009552)
Supplement: S2 Table — We report the minimum and maximum values of the average SNR for each value of the mean polygon number and in each regime of the parameters controlling the communication process. The average is computed over the sets of cell configurations for each value of the number of cells (cf. Fig 5, S2 and S4 Figs). Note that MPN = 6 includes also configurations with 169 and 217 cells. (PDF) [file pcbi.1009552.s010.pdf]

	ISD				NNE	
MPN	Weak-local	Strong-local	Weak-global	Strong-global	Weak	Strong
5.0	[3.28, 4.96]	[3.87, 17.60]	[3.72, 11.23]	[3.89, 18.35]	[2.98, 3.91]	[3.88, 17.12]
5.25	[3.25, 4.96]	[3.85, 17.58]	[3.69, 10.95]	[3.86, 18.30]	[3.00, 4.09]	[3.85, 17.19]
5.5	[3.26, 4.97]	[3.84, 17.53]	[3.67, 10.68]	[3.85, 18.23]	[3.04, 4.25]	[3.84, 17.23]
5.75	[3.24, 4.99]	[3.79, 17.55]	[3.63, 10.45]	[3.80, 18.23]	[3.09, 4.39]	[3.79, 17.32]
6.0	[3.25, 5.04]	[3.82, 21.76]	[3.65, 10.42]	[3.83, 23.17]	[3.14, 4.60]	[3.82, 21.46]
