# Supplementary material for: The role of cell geometry and cell-cell communication in gradient sensing
Source: PLoS Comput Biol. 2022 Mar 14;18(3):e1009552. doi: 10.1371/journal.pcbi.1009552 (PMC8963572; doi:10.1371/journal.pcbi.1009552)
Supplement: S3 Table — We report the values of the CLES−0.5, which varies between −0.5 and 0.5 (see Materials and methods for details), for the comparisons between the distributions of the SNR for different mean polygon number (MPN), shown in Fig 6. A comparison is considered statistically significant if the p-value from a Wilcoxon rank sum test is smaller than 0.05 and |CLES − 0.5| > 0.1. NS indicates that the comparison is not significant, according to this criterion. Communication regimes that are not shown in the table do not have statistically significant results. The last line shows the statistical significance between the extreme values of the mean polygon number interval used in the analysis. (PDF) [file pcbi.1009552.s011.pdf]

MPN	Weak-global (ISD)	Weak (NNE)
5.0 - 5.25	0.14	NS
5.25 - 5.5	0.13	-0.13
5.5 - 5.75	0.13	-0.19
5.75 - 6.0	NS	-0.21
5.0 - 6.0	0.37	-0.42
