# Supplementary material for: The role of cell geometry and cell-cell communication in gradient sensing
Source: PLoS Comput Biol. 2022 Mar 14;18(3):e1009552. doi: 10.1371/journal.pcbi.1009552 (PMC8963572; doi:10.1371/journal.pcbi.1009552)
Supplement: S4 Table — We report the minimum and maximum values of the average SNR for each value of the number of cells and in each regime of the parameters controlling the communication process. The average is computed over the sets of cell configurations for each value of the mean polygon number (cf. Fig 6, S2 and S6 Figs). (PDF) [file pcbi.1009552.s012.pdf]

	ISD				NNE	
# cells	Weak-local	Strong-local	Weak-global	Strong-global	Weak	Strong
7	[3.24, 3.28]	[3.79, 3.87]	[3.63, 3.72]	[3.80, 3.89]	[2.98, 3.14]	[3.79, 3.88]
19	[4.56, 4.61]	[7.00, 7.17]	[6.27, 6.52]	[7.04, 7.21]	[3.77, 4.31]	[6.99, 7.14]
37	[4.91, 5.01]	[10.00, 10.14]	[8.16, 8.56]	[10.11, 10.27]	[3.91, 4.60]	[9.96, 10.06]
61	[4.96, 5.04]	[12.77, 12.89]	[9.34, 9.97]	[13.00, 13.15]	[3.88, 4.59]	[12.67, 12.80]
91	[4.86, 4.96]	[15.27, 15.40]	[9.99, 10.79]	[15.69, 15.81]	[3.85, 4.49]	[15.03, 15.30]
127	[4.71, 4.84]	[17.53, 17.71]	[10.30, 11.23]	[18.23, 18.39]	[3.67, 4.38]	[17.12, 17.56]
