# Supplementary material for: The role of cell geometry and cell-cell communication in gradient sensing
Source: PLoS Comput Biol. 2022 Mar 14;18(3):e1009552. doi: 10.1371/journal.pcbi.1009552 (PMC8963572; doi:10.1371/journal.pcbi.1009552)
Supplement: Attachment — Submitted filename: Fiorentino_Scialdone_Author_response.pdf [file pcbi.1009552.s013.pdf]

Dear Editor,

We would like to resubmit a revised version of our manuscript “The role of cell geometry and cell-cell communication in gradient sensing” (PCOMPBIOL-D-21-01851) for consideration in PLOS Computational Biology.

We thank the three Reviewers for their positive comments and very constructive criticisms, we have taken all of them in account as explained in details below and we believe the manuscript has significantly improved.

In particular, we have extended the discussion about the biological relevance of our models and have performed additional simulations as well as analytical calculations to make our conclusions more robust.

All the changes to the text are listed in the detailed response below and are highlighted in one of the attached files.

We are looking forward to hearing back from you.

Sincerely,
Jonathan Fiorentino and Antonio Scialdone

Response to Reviewers

Reviewer #1

Reviewer #1: In this paper the authors address the question of understanding how cell geometry and cell-cell communication can affect gradient sensing. They use as starting point the local-excitation global-inhibition model (LEGI) and generalise it to investigate the role played by cell number, geometry and long- and short- cell-cell communication in efficiently measuring concentration gradients. To this aim they define two different modes of cell-cell communication, i.e., the nearest neighbour exchange (NNE) where a global reporter allows communication only between nearest neighbour cells, and the Intercellular Space Diffusion (ISD), where the global reporter can be secreted by the cells, diffuse and internalised by other cells. This further ingredient, besides being an elegant way of modelling cell-cell communication, also allows them to span a wide range of interactions according to the diffusion length of the global reporter. Starting from this model and these different interactions schemes, they study the Signal-to-Noise Ratio to quantify the precision of gradient sensing. In summary, their results show that i) according to the type of communication, the precision of gradient sensing can depend on the number of cells. In particular, when the communication is weak and local, smaller systems perform better, whereas when the communication is global and/or strong, larger systems perform better. ii) By varying the tissue geometry, i.e., number of edges per polygon for example, in the regime of fast diffusion in the ISD model, more disordered configurations lead to more precise gradient sensing, whereas ordered structures are beneficial in the regime of nearest neighbours communication.

I find the paper very interesting, well done and well written. I appreciated a lot the extensive explanations about their modelling approach, analysis and obtained results and the literature is well represented. I only have a few minor points which I'd like the authors might clarify and I believe could improve the quality of the manuscript.

Response 1: We thank this Reviewer for his/her very positive comments and we describe below how we addressed the points he/she raised.

i) In the paper, I have noticed that the authors never explicitly mention the variety of differently diffusing morphogens which actually play the same role as their global reporter in different species (see for example Delta-Notch in Drosophila, Nodal-Lefty in zebrafish, Wnt in a number of different species). I think it would be important to discuss this and to also relate their results to some of the morphogens whose diffusion constants have been estimated.

Response 2: Thanks for this suggestion, we have added two paragraphs on this point in the Introduction (Lines 52-66) and Discussion (Lines 395-399) sections.

ii) my main concern lies in the comparison of the Signal-to-Noise Ratios between the ISD and NNE modes of communication in Fig. 5 and 6. In Fig 5 panels A and B it does seem that there's an optimum around 37 cells, which the authors explain in the text. Yet, I think, if it's

possible, it'd be better to extend the simulations further beyond 127 cells to clearly see whether there's a real decrease in the SNR. Same for Fig 5E where instead it seems that the behaviour of SNR tends to saturation. In Fig 6, instead, in particular panel B and E, I believe it would be better to extend the mean polygon number to a wider range (e.g., between 3 and 8?) as well as make sure that the parameters used in the model as optimal in order to observe the highest variations in the plots. At the moment, in fact, the fold decrease/increase in panels E and B is quite small to make actual claims in my opinion (or is there a point which I am missing?).

Response 3: We thank the reviewer for the comment, which allows us to strengthen and clarify our results on the Signal-to-Noise Ratios (SNRs) in the NNE and ISD models.

We have now extended the simulations to configurations with N=169 and N=217 cells in the new Figure 5 (included also below). As expected, in panels A and B we observe a significant decrease of the SNR even beyond N=127 cells, while panel E shows a saturation of the SNR.

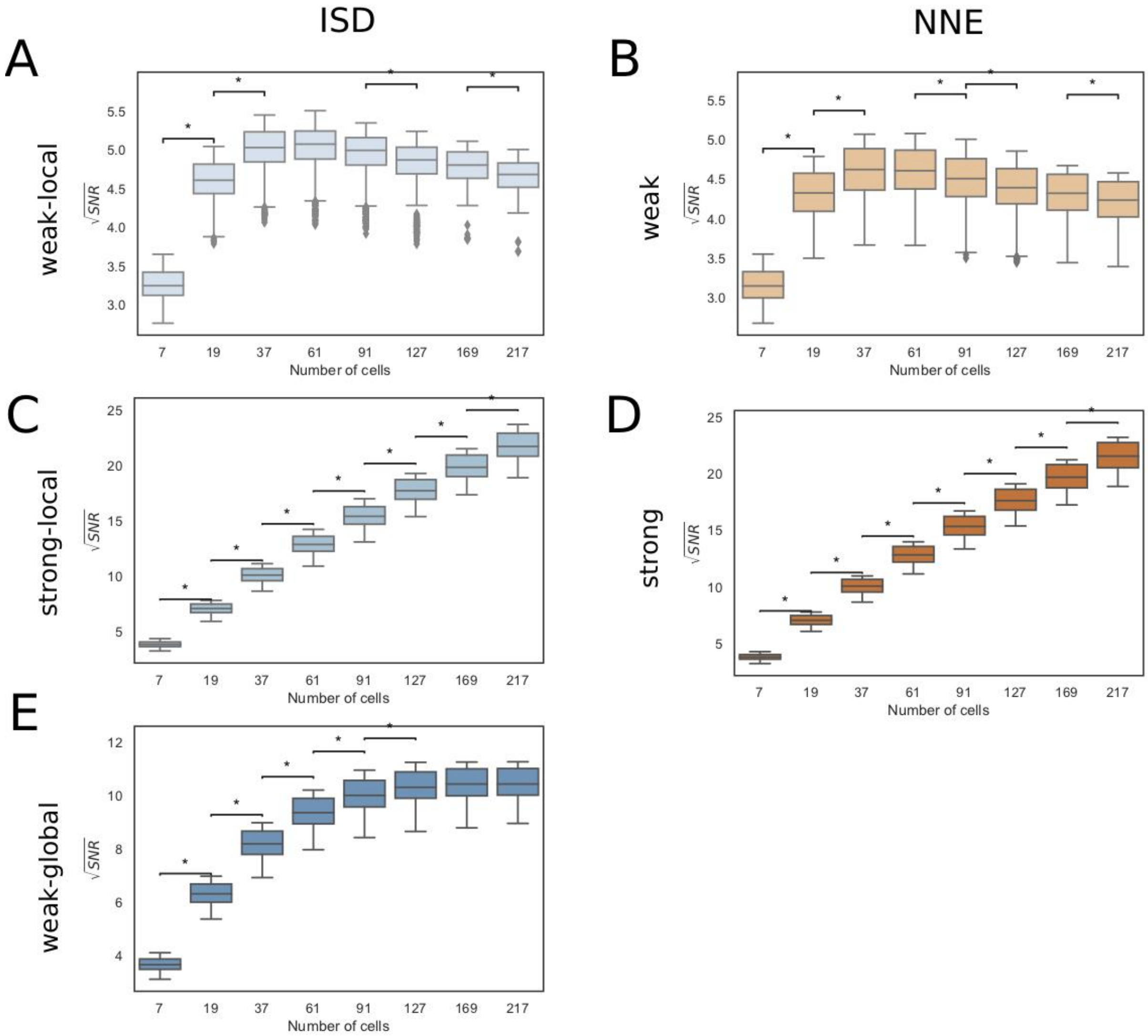

Regarding the values of the mean polygon number, in our simulations we have kept this number within the range observed in the experiments (see, e.g., references [6,50-52] in the

manuscript), and the simulated configurations are typically mixtures of cells with different numbers of sides (between 3 and 6), as observed in the experimental studies mentioned above. While heptagonal and octagonal cells are present (especially in Drosophila), they occur less often than pentagonal and hexagonal cells, hence they are unlikely to have an appreciable effect on the SNR. Moreover, while the fold-change is relatively small (albeit significant) between successive values of the mean polygon number, there is a clear increasing or decreasing trend and the difference between SNR values at, e.g., mean polygon number 5 and 6 are strongly statistically significant (the common language effect sizes or CLES is -0.42 for Figure 6B and 0.37 for Figure 6E; note that CLES varies between -0.5 and 0.5).

We added a sentence on this point in the manuscript at Lines 301-303 and we now report also the common language effect sizes (CLES) computed between the mean polygon number 5 and 6, relative to Figure 6B and 6E, in Supplementary Table S3.

Also, I would honestly move Fig S3-S4 directly in the Main Text as in my opinion they summarise most of the results and are amazingly clear. To better highlight the results, I would also use the same scale across all the different panels.

<u>Response 4:</u> We agree with the Reviewer that Figure S3 and S4 (S4 and S6 in the revised version of the manuscript) nicely summarize the results, but, for the sake of conciseness, we would prefer to keep them in the supplementary material.

In these panels, we have used different scales to emphasize the differences in the trends of SNR across the different regimes. However, we think the Reviewer is right in pointing out that comparing the SNR values across regimes is also interesting and we have added a table reporting the min and max SNR in each regime (new Supplementary Tables S2 and S4).

iii) Reference 38 appears to miss the authors.

<u>Response 5:</u> We corrected the reference (it is reference 53 in the revised version of the manuscript), thanks for catching it!

Reviewer #2

Reviewer #2: In this manuscript, the Authors propose and computationally characterize a model of collective gradient sensing for epithelial cell monolayers, where distant cells communicate with each other by secreting a molecular reporter which can diffuse along cell-cell contacts.

The model belongs to the class of ‘local excitation, global inhibition’ (LEGI) models, and generalizes a similar model previously introduced in 2016 by Levchenko and collaborators.

The simple idea behind LEGI models is that a cell, or, in these more recent proposals, a group of cells, would locally produce a molecular factor proportional to a sensed external gradient, while a second, rapidly diffusing molecular factor would ‘compute’ an average of the external signal, to be subtracted to the signal itself, so that the resulting (positive and negative) differences could then be amplified to give opposing responses.

In the approach proposed here, the role of the global inhibitor is played by a hypothetical molecular reporter which is secreted by the epithelial cells along cell-cell contacts, diffuses along cell-cell contact lines, and is later internalized either by nearest-neighbor cells, or by more distant cells.

My greater perplexity about this work regards this specific hypothesis, as I don't know of evidence supporting the idea that a molecular reporter would travel specifically in the quasi one-dimensional region of cell-cell contacts. In their Introduction, the Authors name as possible candidates small molecules such as ATP, calcium ions and NO; soluble ligands that bind EGFR; and extracellular vesicles. However, small molecules are not likely to be confined to the region of cell-cell contacts. Perhaps, one may imagine that tight junctions could confine the diffusion of larger molecular reporters to the quasi one-dimensional cell-cell contact region. Or, that confinement could be implemented by rapid association and dissociation to/from receptors specifically concentrated along cell-cell contacts. But in any case, this main conceptual difficulty should be discussed. Either the Authors know about, or at least suspect the existence of possible biological realizations of their abstract model, and in that case, the paper should be integrated with a discussion of these examples. Or their model is purely speculative, and in that case the speculative character of the hypotheses should be more clearly stated.

Response 1: We thank the Reviewer for the constructive criticism, we agree that the hypotheses at the basis of the intercellular space diffusion (ISD) model were not thoroughly discussed in our manuscript.

Probing the diffusion of molecules in the lateral intercellular space is experimentally challenging, but there is specific evidence of processes that could confine the diffusion of molecules in the lateral intercellular space. For example, molecules like Wnt proteins can dynamically associate/dissociate from cell membranes and diffuse on them [54], and tight junctions can significantly reduce the outflux of molecules with a size greater than ~3.5 angstrom [55] (as a reference, the size of ATP is ~14 angstrom and Wnt proteins ~60-70 angstrom). We now discuss these in the revised text (lines 385-394).

While these observations suggest that it is plausible that molecular reporters could stay mostly confined and diffuse in the lateral intercellular space (LIS), we performed additional

analyses to show that our model does not require a total confinement of the global reporter in the LIS. More specifically, we introduce in our model an "escape rate" for the global reporter (see Supplementary Text) and estimate how cellular communication is affected in the ISD model.

We showed that an escape rate <20% of the internalization rate has little effect on the probability of molecular exchanges between cells (see new Supplementary Figure S1), leaving our main conclusions substantially unaffected. Larger escape rates could hinder cell communication and would eventually make the ISD model more similar to the nearest-neighbour exchange (NNE) model.

These new analyses support the plausibility of our model and also provide more precise constraints on the properties of the global reporter in the ISD model.

Secondly, other ways (for instance, mechanical) of introducing long-range intercellular communication for collective gradient sensing have been proposed: a discussion of these alternatives would therefore be in order.

Response 2: This is a very important point, thanks for raising it. We have added the following paragraph to the Discussion section of our manuscript (Lines 405-414):

“Moreover, other ways of introducing long-range cell-cell communication are possible: examples include the exchange of molecules through cellular protrusions, such as filopodia or cytonemes [57-58], or other cellular channels, such as epithelial bridges [59] and tunnelling nanotubes [60-61]. Our model could account for this means of intercellular communication, replacing the diffusion in the lateral intercellular space with that through cellular protrusions. In this case, probing different cell configurations varying the mean polygon number would amount to consider different distributions of the number of connections of a cell with neighbours of different order. Finally, mechanical ways of obtaining long-range communication are possible, as observed in the Drosophila ovary with the presence of mechanical feedback through cadherins [62].”

Apart from these main difficulties, the paper is well conceived and well written. The model is clearly exposed and thoroughly characterized, the computational and statistical methods used are sound. In the framework of the model, it is shown that long-range communication would be beneficial to collective gradient sensing, especially when low levels of molecular reporters are secreted. Additionally, ‘disordered’ epithelial monolayers are shown to be able to sense gradients with a marginally higher signal-to-noise ratio, if compared to perfectly ordered (purely hexagonal) monolayers.

In conclusion, I do not think that in its present version this manuscript has the level of significance for the discipline which is required for publication in PLOS Computational Biology. That would change in my opinion if the Authors were able to convincingly make the case for the plausibility of their model.

Response 3: We thank the Reviewer for these very positive comments and we hope that the additional analyses and discussion addressed his/her concern.

Minor observations:

- Sect. 3.3: it is shown that in the 'weak-global' regime, the SNR is higher in configurations with lower mean polygon number; this is interesting, and it would be nice to have an intuitive understanding of this effect; perhaps it is related to the fact that if the area is kept fixed, the perimeter of polygons with a smaller number of edges is smaller?

Response 4: We also think this result is interesting. We believe it is related to the presence of higher-order vertices which, in turn, allows the global reporter to more efficiently reach cells at larger distances, thus increasing the efficiency of long-range communication (see Lines 322-325 and Figure 6F). However, as the Reviewer suggests, the smaller perimeter of polygons with lower number of edges (see new Supplementary Figure S5) might also contribute to this effect, as we now state in Line 325-327 of the Result section.

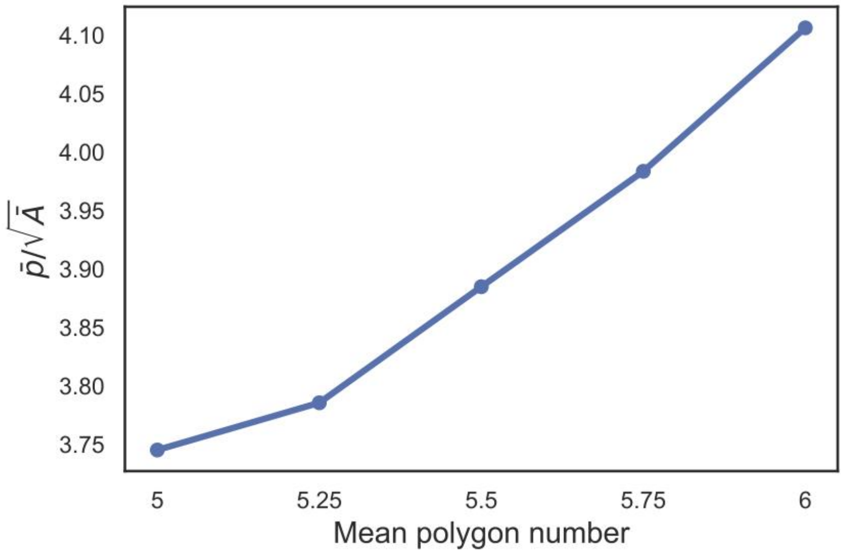

- Fig. 6.F: it would be useful to explain the rationale for dividing the distances by the average edge length.

Response 5: We divided the distances by the average edge length, which is l_E= 7.1 micron, so that the x-coordinate indicates approximately the order of neighbouring cells. We clarified this point in the caption of Fig 6F.

- Eq. 3: there is a wrong index (n in the place of k); actually, it would be more clear to have everywhere (in Eqs. 3,4 and in the text) the couple of indices k,k'; or, alternatively, n,n'.

Response 6: We thank the Reviewer for noticing this. We corrected the index in Eq. (3) and we now use the pair of indices n, n' in the equations and in the text.

- Eq. 12: a differential of tau seems to be missing in the second integral; also, the use of the letter d for both the distance and the differential is confusing; perhaps it could be convenient

to use s instead; it could also be useful to have a reference (for instance, Redner 2001) for the first-passage time formula used.

Response 7: We added the differential of tau in Eq. (12) and used s for the distance, as suggested by the Reviewer. We also added the suggested reference (ref. 49) for the first-passage time distribution.

- Fig. 3, caption: ‘edges represent . . . ’; perhaps: ‘edge thickness represent . . . ’? it is not clear how exactly the different weights are represented in the weighted graph.

Response 8: We changed the caption of Fig. 3 to clarify this point. The edge thicknesses represent weights, while continuous vs dashed edges represent nearest-neighbours vs non-nearest-neighbours connections, respectively.

- Fig. 1, caption: there is a mention of ‘edge cells’, but the notion of ‘edge cells’ has not been discussed previously.

Response 9: We clarified the definition of edge cell in the caption of Fig. 1.

- The year is missing in Ref. 15.

Response 10: We corrected the reference, thanks for catching it!

Reviewer #3

Reviewer #3: Re: Review of "The role of cell geometry and cell-cell communication in gradient sensing" by Jonathan Fiorentino and Antonio Scialdone.

The paper investigates how groups of cells collectively sensing shallow gradients of external systems. Cellular communication is modelled using a local-excitation global-inhibition (LEGI) model. A LEGI model has two components: (1) a local reported confined to a cell representing a local measurement of the concentration, and (2) global reported molecules produced in response to the external signal, which is exchanged with neighbouring cells. Previous work includes using LEGI models to study collective sensing of external gradients in one-dimensional and very simple two-dimensional geometries. This paper extends these results to more complicated (and realistic) two-dimensional geometries. The authors investigate how the signal-to-noise ratio (SNR) depends on the number of cells, geometry, local vs. global communication mechanism, and strength of nearest neighbour communication.

In more detail, two different models of cell-cell communication are considered: (1) nearest neighbour exchange of the global reporter (short-ranged), and (2) intercellular space diffusion (ISD) of the global reported on the cell-cell interfaces (long-ranged). For the ISD model the nonlocal exchange rates between cells are estimated by computing 5000 trajectories of diffusing global reported molecule (for each geometry).

Result Summary

The authors find that:

- Smaller systems (with respect to cell number) tend to have higher SNR when cell communication is weak or local.

- Larger systems have higher SNR when cell communication is global (with ISD) and/or strong.

- The NNE / ISD have similar SNR in the strong-local regime i.e. when the reporter is mostly local and efficiently exchanged between cells.

- Finally the authors investigated the effect of geometry on the SNR. Interestingly, for the ISD model more irregular geometries have higher SNR.

Conclusion

The results of this study are particularly significant to epithelial tissues which feature dynamically changing geometries, since it is currently unknown how such geometric arrangement affect

collective gradient sensing. The insights of this study are of high interest to modellers of epithelial tissues (e.g. cellular Potts models, vertex models etc.). In particular, those modelers

considering processes depending on external gradient sensing (e.g. development). Finally the study is interesting, well written and well presented. The code for studies' results appear to be completely available on github. I recommend this study for publication.

Response 1: We are very happy this Reviewer finds our results interesting and well presented, and that he/she endorses the publication of our manuscript.

We answer below his/her comments.

Specific Comments:

On line 103 you define the signal to noise ratio as your primary objective.

- It might be beneficial for the reader to define ``good'' or ``bad'' values of the SNR, so that a reader has some reference points in mind when encountering the models results.

Response 2: This is an interesting suggestion. However, it is not trivial to decide what value of SNR could be considered “good” or “bad”, since it highly depends on the features of the system at study. For example, upper bounds of the SNR in 1-dimensional systems were derived under specific approximations (see reference [19] in our manuscript). Similar estimations could be made in our more complex 2-dimensional systems, but the set of assumptions would be rather arbitrary and might distract from the main conclusions that rely on the comparisons of SNR across different cell geometries, system size and mechanisms of cell communication.

- I'm confused by the line "To compute it, we use the formula obtained in [18]". What is it here? Is it the SNR? Because isn't the SNR computed using equations (5) and (6)?

Response 3: We agree with the Reviewer that the sentence was unclear, and we have replaced it with the following one (Lines 121-122): “To compute it, we use Eqs. (5) and (6), following the derivation presented in [18]”.

Minor comments / questions:

Line 22: What do you mean with physical limits?

Response 4: We mean the limits imposed to sensory precision by the presence of cellular communication, which were derived in reference [19]. These limits depend on a number of factors, like the properties of the external signal, the cell size and the typical length scale of cellular communication (which in turn depends on the kinetic rates of the LEGI global reporter).

We clarified this point in the text at Lines 22-24.
